# Crosstalk between glial and glioblastoma cells triggers the “go-or-grow” phenotype of tumor cells

**DOI:** 10.1186/s12964-017-0194-x

**Published:** 2017-10-02

**Authors:** Ana Isabel Oliveira, Sandra I. Anjo, Joana Vieira de Castro, Sofia C. Serra, António J. Salgado, Bruno Manadas, Bruno M. Costa

**Affiliations:** 1 Life and Health Sciences Research Institute (ICVS), School of Medicine, Campus de Gualtar, University of Minho, 4710-057 Braga, Portugal; 2 ICVS/3B’s - PT Government Associate Laboratory, Braga/Guimarães, Campus de Gualtar, University of Minho, 4710-057 Braga, Portugal; 3 CNC - Center for Neuroscience and Cell Biology, University of Coimbra, 3004-504 Coimbra, Portugal; 4 Faculty of Sciences and Technology, University of Coimbra, Coimbra, Portugal

**Keywords:** Glioblastoma, Glial cells, Tumor microenvironment, Secretome, Paracrine effect

## Abstract

**Background:**

Glioblastoma (GBM), the most malignant primary brain tumor, leads to poor and unpredictable clinical outcomes. Recent studies showed the tumor microenvironment has a critical role in regulating tumor growth by establishing a complex network of interactions with tumor cells. In this context, we investigated how GBM cells modulate resident glial cells, particularly their paracrine activity, and how this modulation can influence back on the malignant phenotype of GBM cells.

**Methods:**

Conditioned media (CM) of primary mouse glial cultures unexposed (unprimed) or exposed (primed) to the secretome of GL261 GBM cells were analyzed by proteomic analysis. Additionally, these CM were used in GBM cells to evaluate their impact in glioma cell viability, migration capacity and activation of tumor-related intracellular pathways.

**Results:**

The proteomic analysis revealed that the pre-exposure of glial cells to CM from GBM cells led to the upregulation of several proteins related to inflammatory response, cell adhesion and extracellular structure organization within the secretome of primed glial cells. At the functional levels, CM derived from unprimed glial cells favored an increase in GBM cell migration capacity, while CM from primed glial cells promoted cells viability. These effects on GBM cells were accompanied by activation of particular intracellular cancer-related pathways, mainly the MAPK/ERK pathway, which is a known regulator of cell proliferation.

**Conclusions:**

Together, our results suggest that glial cells can impact on the pathophysiology of GBM tumors, and that the secretome of GBM cells is able to modulate the secretome of neighboring glial cells, in a way that regulates the “go-or-grow” phenotypic switch of GBM cells.

**Electronic supplementary material:**

The online version of this article (10.1186/s12964-017-0194-x) contains supplementary material, which is available to authorized users.

## Background

Glioblastoma (GBM), the most common of all malignant brain and central nervous system (CNS) tumors in adults [1], is characterized by a poor outcome and limited therapeutic options. Standard GBM management involves surgical resection followed by radiotherapy with concomitant and adjuvant chemotherapy with temozolomide (TMZ), but in most cases GBM rapidly relapses [2]. Hence, the treatment remains mostly palliative, with a median overall survival of only 15 months after diagnosis [3]. The difficulty in effectively treating GBM patients is largely due to the heterogeneous nature of these tumors [4] and also to the complex network of interactions that they establish with the tumor microenvironment (TME) [5]. Therefore, the TME has been suggested to play vital roles in controlling the course of GBM and influencing its treatment response [6].

In brain tumors, the microenvironment consists of a complex network of interactions between a large variety of glioma-associated stromal cells, including, for example, astrocytes, endothelial cells, and infiltrating inflammatory cells like microglia, among others [5]. The crosstalk between these cells has been shown to favor tumor growth, invasiveness and therapy resistance, driven by paracrine signals and disseminated by secreted factors [7, 8]. In particular, the role of microglia cells and astrocytes in glioma progression and aggressiveness has been studied (reviewed in [9]), confirming the existence of paracrine interactions between GBM cells and TME.

Astrocytes are the most abundant non-neuronal cells in the brain (consisting of approximately 50% of the human brain volume), and are therefore likely to establish myriad direct contacts with tumor cells, potentially influencing glioma pathophysiology. Indeed, the invasiveness of glioma cancer stem cells was shown to be increased in the presence of astrocytes or astrocytes-derived conditioned media (ACM) [10]. Similarly, the migration capacity of human and murine glioma cell lines is increased in the presence of ACM [11], and GDF-15, known to be upregulated in reactive astrocytes [12], was found to increase proliferation of glioma cells [13]. Taken together, these findings suggest a role for the secretome of astrocytes in the processes of glioma initiation and progression. Importantly, a cell secretion is a highly dynamic and sensitive process that can change dramatically and rapidly, such as in a disease context [14]. Thus, while the secretome of naive astrocytes has already been mapped [15–17], its characterization in the context of exposure to glioma cells is still missing. In this study, we have investigated the bidirectional communication between GBM and glial cells. Our data suggest that factors secreted by GBM cells are capable of modulating the secretome of glial cells leading to the upregulation of several proteins related to cellular homeostasis, cell adhesion and defense response. Additionally, we found that this modulation functions in a paracrine fashion to regulate the “go-or-grow” phenotypic switch of GBM cells.

## Methods

### Mouse glioma cell line

The murine glioma cell line GL261 was maintained in Dulbecco’s Modified Eagle Medium (DMEM, Biochrom) with 10% fetal bovine serum (FBS, Biochrom) and 1% penicillin- streptomycin (Gibco). Incubations were performed at 37 °C in a humidified atmosphere containing 5% CO_2_.

### Primary cultures of cortical glial cells

Cortical glial cells were isolated from the brain of P5-P7 C57Bl/6 wild type pups. Brain macro-dissection was performed in ice-cold HBSS, under a conventional light microscope. Upon dissection, cortices were cut into smaller fragments and incubated for 30 min at 37 °C in HBSS supplemented with 0.6% trypsin and 750 units of DNase I (both from Sigma). The digested tissue fragments were washed twice with DMEM supplemented with 10% FBS and 1% penicillin-streptomycin and mechanically dissociated through a 5 mL pipette and a P1000. A single-cell suspension in complete DMEM was obtained and cells were plated according to the following experiments. For conditioned media experiments, 2 × 10^6^ cells were seeded in T75 flasks; for immunocytochemistry, cells were plated on coverslips coated with poly-D-lysine (Sigma-Aldrich) at a density of 25,000 cells/cm^2^; and for viability assay (MTT) cells were seeded at the same density in culture wells. The culture medium was changed every 2–3 days and glial cells were maintained in culture for 15–18 days, at 37 °C in a humid atmosphere (5% CO_2_).

### Conditioned media collection and experiments

To obtain conditioned medium (CM) from GL261, cells were plated at a density of 8600 cells/cm^2^ and allowed to grow for 72 h. Following this, cells were washed 2 times with PBS and one with serum-free DMEM and the culture medium was replaced by serum-free DMEM. The CM was collected after 24 h and filtered through 0.22 μm pore size filters. Upon collection, CM was snap-freeze in liquid nitrogen and kept at −80 °C until the day of experiments.

For glial cells CM production, cells were allowed to grow until they reach a monolayer (15–18 days) and then washed twice with PBS and once with serum-free DMEM. Flasks were randomly distributed in two groups: one received serum-free DMEM for 72 h (unexposed) and the other GL261 CM (exposed). In both groups, the medium was replaced after 48 h by fresh medium (serum-free DMEM or GL261 CM, respectively). After 3 days, glial cells growing in serum-free DMEM (unprimed) and glial cells exposed to GL261 CM (primed) were washed as previous and serum-free DMEM was added to both groups. Twenty-four hours thereafter, CM from unprimed and primed glial cells was collected and stored as described above for GL261 CM. Glial cells were then submitted to viability assay and immunocytochemistry.

### Cell viability assays

Cell viability of glial cells unexposed and exposed to GL261 CM was quantified using 3-(4,5-Dimethylthiazol-2-yl)-2,5-Diphenyltetrazolium Bromide assay (MTT, Invitrogen). Cells were exposed to 0.5 mg/mL of MTT in PBS for 2 h in a humidified atmosphere at 37 °C and 5% CO_2_. The formazan was then solubilized in acidified isopropanol (0.04 M HCl in absolute isopropanol) and the optical density was determined at 570 nm.

For GL261 glioma cells viability, 5 × 10^4^ cells were plated/well in a 6-well plate and incubated at 37 °C and 5% CO_2_ for 72 h. Following this, cells were washed and the culture medium was replaced by CM from unprimed or primed glial cells. After 48 h of incubation, total viable cells were determined by trypan blue exclusion assay (Trypan Blue Solution, 0.4%, Gibco).

### Immunocytochemistry

Glial cells unexposed or exposed to GL261 CM were fixed in 4% paraformaldehyde at room temperature (RT) for 30 min and permeabilized with 0.3% Triton X-100 in PBS for 5 min. Cells were then blocked with 10% FBS in PBS for 1 h at RT followed by the incubation with anti-Glial Fibrillary Acidic Protein (GFAP, Dako), diluted 1:1000 in 10% FBS in PBS, for 1 h at RT. Cells were washed with 0.5% FBS in PBS and incubated for 1 h at RT with Alexa Fluor® 594 conjugate (Invitrogen) diluted in 10% FBS in PBS (1:1000). Finally, cells were washed with 0.5% FBS in PBS and the glass coverslips were mounted in VECTASHIELD® Mounting Medium with DAPI (Vector Laboratories). Fluorescence analysis and image capture were performed under an Olympus BX-61 Fluorescence Microscope (Olympus).

### RNA extraction, cDNA synthesis and qRT-PCR

Total RNAs from glial cells unexposed or exposed to GL261 CM were extracted with Trizol (Invitrogen) according to the manufacturer’s instructions. cDNA synthesis was performed using 1 μg of total RNA with High Capacity cDNA Reverse Transcription Kit (Applied Biosystems). Gene-specific mRNA levels were assessed by quantitative real-time PCR (qPCR) in a real-time thermocycler (CFX96; Bio-Rad) using Fast SYBR Green (Qiagen) or PowerUp SYBR (Applied Biosystems; for *p21* gene) according to the manufacturer’s instructions, by the 2^-ΔΔCt^ method. The list of primers used and the PCR conditions can be found in Additional file 1: Table S1.

### Sample preparation for proteomics analysis

Glial cells CM (unprimed and primed) spiked with the recombinant protein *mal*E-GFP (to be used as internal standard) was firstly concentrated using a Vivaspin® Turbo 15 sample concentrator (5 kDa; Sartorius) by ultracentrifugation at 4000×g. Concentrated CM was precipitated with Trichloroacetic acid (TCA) - Acetone [18]. The washed pellets were ressuspended in 40 μL of 2× Laemmli buffer (BioRad), aided by ultrasonication and denaturation at 95 °C [19]. Ten microlitres of each replicate (in a total of 4 replicates per condition) were used to create a pooled sample for protein identification. After denaturation, samples were alkylated with acrylamide and subjected to gel digestion using the short-GeLC approach [20]. The entire lanes were sliced into 3 parts and each part was sliced in small pieces and processed. Gel pieces were destained, dehydrated and re-hydrated in 75 μL of trypsin (0.01 μg/μL solution in 10 mM ammonium bicarbonate) for 15 min, on ice. After this period, 30 μL of 10 mM ammonium bicarbonate were added and in-gel digestion was performed overnight (ON) at RT. After the digestion, the formed peptides were extracted from the gel pieces and the peptides extracted from the three fractions of each biological replicate were combined into a single sample for quantitative analysis. All the peptides were dried subjected to SPE using OMIX tips with C18 stationary phase (Agilent Technologies) as recommended by the manufacture. Eluates were dried and ressuspended with a solution of 2% ACN and 0.1% FA.

### Protein quantification by SWATH-MS

Samples were analyzed on a Triple TOF™ 5600 System (ABSciex®) in two phases: information-dependent acquisition (IDA) of the pooled samples and SWATH-MS acquisition of each individual sample. Peptides were resolved by liquid chromatography (nanoLC Ultra 2D, Eksigent®) on a MicroLC column ChromXP™ C18CL (300 μm ID × 15 cm length, 3 μm particles, 120 Å pore size, Eksigent®) at 5 μL/min with a multistep gradient: 0–2 min linear gradient from 5 to 10%, 2–45 min linear gradient from 10% to 30% and, 45–46 min to 35% of ACN in 0.1% FA. Peptides were eluted into the mass spectrometer using an electrospray ionization source (DuoSpray™ Source, ABSciex®) with a 50 μm internal diameter (ID) stainless steel emitter (NewObjective).

Information dependent acquisition, experiments were performed for each pooled sample and the mass spectrometer was set to scanning full spectra (350–1250 *m/z*) for 250 ms, followed by up to 100 MS/MS scans (100–1500 *m/z* from a dynamic accumulation time – minimum 30 ms for precursor above the intensity threshold of 1000 – in order to maintain a cycle time of 3.3 s). Candidate ions with a charge state between +2 and +5 and counts above a minimum threshold of 10 counts per second were isolated for fragmentation and one MS/MS spectra was collected before adding those ions to the exclusion list for 25 s (mass spectrometer operated by Analyst® TF 1.7, ABSciex®). Rolling collision was used with a collision energy spread of 5. Peptide identification and library generation were performed with Protein Pilot software (v5.1, ABSciex®), using the following parameters: i) search against a database composed by *Mus musculus* from SwissProt (release at December 2015), and *mal*E-GFP; ii) acrylamide alkylated cysteines as fixed modification; iii) trypsin as digestion type. An independent False Discovery Rate (FDR) analysis using the target-decoy approach provided with Protein Pilot software was used to assess the quality of the identifications and positive identifications were considered when identified proteins and peptides reached a 5% local FDR [21, 22].

For SWATH-MS based experiments, the mass spectrometer was operated in a looped product ion mode [23] and the same chromatographic conditions used as in the IDA run described above. The SWATH-MS setup was designed specifically for the samples to be analyzed (Additional file 2: Table S2), in order to adapt the SWATH windows to the complexity of the set of samples to be analyzed. A set of 60 windows of variable width (containing 1 *m/z* for the window overlap) was constructed covering the precursor mass range of 350–1250 *m/z*. A 250 ms survey scan (350–1500 *m/z*) was acquired at the beginning of each cycle for instrument calibration and SWATH MS/MS spectra were collected from 100 to 1500 *m/z* for 50 ms resulting in a cycle time of 3.25 s from the precursors ranging from 350 to 1250 *m/z*. The collision energy for each window was determined according to the calculation for a charge +2 ion centered upon the window with variable collision energy spread (CES) according with the window.

A specific library of precursor masses and fragment ions was created by combining all files from the IDA experiments, and used for subsequent SWATH processing. Libraries were obtained using Protein Pilot™ software (v5.1, ABSciex®) with the same parameters as described above. Data processing was performed using SWATH™ processing plug-in for PeakView™ (v2.0.01, ABSciex®) as described in [20]. After retention time adjustment using the *mal*E-GFP peptides, up to 15 peptides, with up to five fragments each, were chosen per protein, and quantitation was attempted for all proteins in library file that were identified below 5% local FDR from ProteinPilot™ searches. Peptides’ confidence threshold was determined based on a FDR analysis using the target-decoy approach and those that met the 1% FDR threshold in at least three of the four biological replicates were retained, and the peak areas of the target fragment ions of those peptides were extracted across the experiments using an extracted-ion chromatogram (XIC) window of 4 min with 100 ppm XIC width.

The levels of the mouse proteins were estimated by summing all the filtered transitions from all the filtered peptides for a given protein (an adaptation of [24]) and normalized to the internal standard (*mal*E-GFP).

The MS proteomics data have been deposited to the ProteomeXchange Consortium [25] via the Proteomics Identifications (PRIDE) partner repository with the dataset identifier PXD006007.

### Functional annotation

Functional clustering of the differentially secreted proteins was performed using the Database for Annotation, Visualization and Integrated Discovery (DAVID) and displayed in Kyoto Encyclopedia of Genes and Genomes (KEGG) and Gene Ontology (GO).

### Cell death

GL261 cell line was plated at an initial density of 1.0 × 10^5^ cells per T25 flask in 3 mL of complete DMEM. After 72 h, cells were washed and the medium was replaced by CM from unprimed or primed glial cells. Cell death was evaluated after 48 h of CM exposure by Annexin V-FITC staining, according to the manufacturer’s instructions (BD Biosciences), followed by flow cytometry analyses. A total of at least 10,000 events were acquired. Results were analyzed by FlowJo Software (version 7.6).

### Migration assay

GL261 cells (7.5 × 10^4^) were seeded in 12-well plates and incubated for 72 h. Monolayer cells were scraped with a plastic pipette tip creating a gap in the monolayer and then incubated with unprimed or primed glial cells CM. Gap closure was evaluated every 24 h for a total of 48 h. The relative migration distance was calculated by the following formula: percentage of gap closure (%) = 100 - (B*100)/A, where A is the width of cell gaps before incubation (0 h), and B is the width of cell gaps after incubation.

### Western blot analysis

GL261 cells were seeded in T25 flasks at a density of 1 × 10^5^ cells per flask and allowed to grow for 72 h. Cells were then washed and the medium replaced by CM from glial cells (unprimed and primed). After 48 h, cells were trypsinized, washed with PBS and lysed on ice for 20 min in lysis buffer [50 mM Tris pH 7.5, 150 mM NaCl, 5 mM EDTA, 1 mM Na_3_VO_4_, 10 mM NaF, 10 mM NaPyrophosphate, 1% NP-40 and 1× Protease inhibitors cocktail (Roche)]. Western blotting was performed using standard 12% SDS-PAGE gel, loading 20 μg of protein per lane. Proteins were transferred onto Hybond nitrocellulose membranes (GE Healthcare), blocked with 5% non-fat milk in TBS + 0.1% Tween-20 (TBS-T) and incubated ON at 4 °C with primary antibody. The following antibodies were used: Phospho-p44/42 MAPK [Erk1/2, Thr202/Tyr204, (1:1000)], Phospho-SAPK/JNK [Thr183/Tyr185, (1:2000)], Phospho-Akt [Ser473, (1:1000)], p44/42 MAPK [Erk1/2, (1:1000)], SAPK/JNK (1:1000), Akt (1:2000), all from Cell Signaling Technology, and α-tubulin (1:1000, Santa Cruz Biotechnology). HRP conjugated goat anti-mouse and goat anti-rabbit (Santa Cruz Biotechnology) were used as secondary antibodies. Subsequently ECL detection (SuperSignal® West Femto, Thermo Scientific) was performed. Band intensity was quantified using Image J software.

For glial cells protein assessment, cells were washed with PBS immediately after CM collection and stored at −80 °C. For protein isolation, cells were lysed on ice for 20 min in lysis buffer [50 mM Tris-HCl, pH 7.4; 1% (*v*/v) Igepal; 0.25% (v/v) sodium-deoxycholate; 150 mM NaCl; 1 mM DTT; 1 mM EDTA, Complete Mini protease inhibitor mixture and Complete Mini phosphatase inhibitor mixture (Roche)]. Western blotting was performed using 12.5% SDS-PAGE gel, loading 30 μg of protein per lane. Proteins were transferred onto low fluorescence polyvinylidene fluoride (PVDF) membranes (TBT RTA TRANSFER KIT, Bio-Rad). The following antibodies were used: p16, p21 and GLB1 (all from Abcam), and Lamin B1 (Santa Cruz Biotechnology). Alkaline phosphatase conjugated anti-rabbit and anti-goat were used as secondary antibodies. Protein-immunoreactive bands were developed using the “Enhanced Chemifluorescence (ECF) detection system” (GE Healthcare) and visualized in a Molecular Imager FX System (Bio-Rad). For determination of the total intensity of the sample loaded, the membrane was further stained using the ServaPurple Total Protein Staining kit (SERVA Electrophoresis GmbH) according with the manufacturer’s instructions. After staining, the membrane was dried and the signal was visualized in a Molecular Imager FX System (Bio-Rad) using the SYPRO Red filter. The adjusted volumes (total intensities in a given area with local background subtraction) for each band and the total intensity of each lane were obtained using the Image Lab software (version 5.1, Bio-Rad).

### Statistical analysis

For the MS data analysis, primed/unprimed ratios were calculated per each replicate and Grubbs test was used to remove outliers. One-sample Student’s *t*-test against a theoretical value of one was applied to the ratios using SPSS 21.0 (IBM SPSS Statistics, IBM®). For in vitro assays, single comparisons between the different conditions studied were done using a paired Student’s t test. Statistical analysis was done using Graph Pad Prism version 6. Data are presented as mean ± SEM. The level of significance in all the statistical analysis was set at *p* < 0.05.

## Results

### CM from GBM cells does not affect glial cell viability or cellular composition

**Fig. 1:**
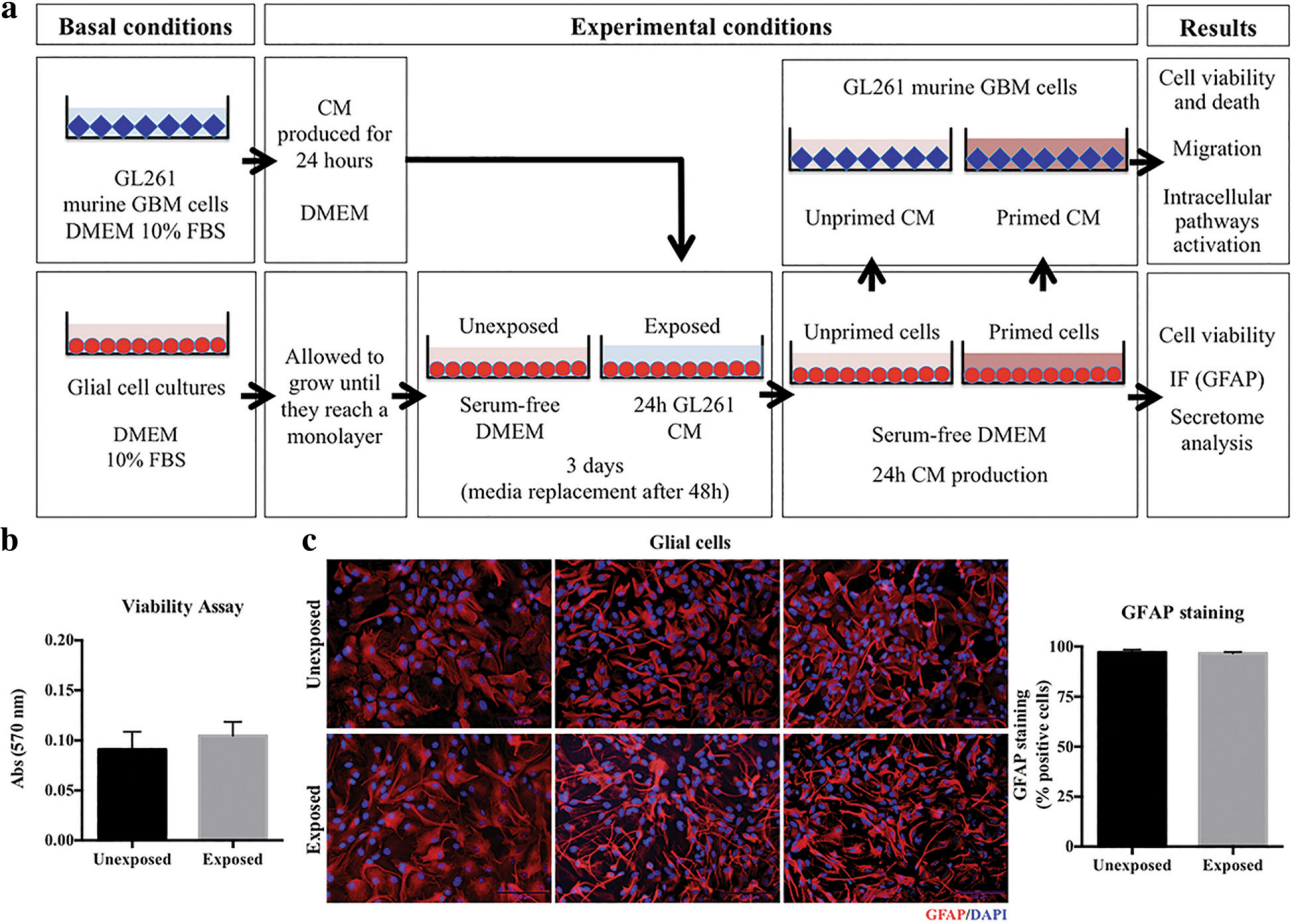
Experimental design and glial cells phenotype in the presence of GBM cells secretome. **a.** Diagram of glial cells-priming experimental setup. GL261 cells were cultured for 24 h to generate tumor-conditioned media (shown in *blue*). Glial cells were incubated for 3 days in GL261-conditioned medium (i.e., exposed) or non-conditioned medium (i.e., unexposed). Fresh non-conditioned medium was then added to glial cells (unprimed and primed). After 24 h, conditioned medium from unprimed (*light red*) or primed (*dark red*) glial cells was added to GL261 murine GBM cells. Glial cells were analyzed for cell viability and GFAP marker and proteomics characterization of the secretome. Functional and mechanistic assays were performed in GL261 cells. **b.** MTT cell viability assay in glial cells unexposed or exposed to GL261 CM. **c.** Immunocytochemistry staining for GFAP (left) and quantification of GFAP-positive cells (right) in glial cells unexposed or exposed to GL261 CM. Abbreviations: GBM, glioblastoma; DMEM, Dulbecco’s Modified Eagle Medium; FBS, Fetal Bovine Serum; CM, conditioned media; IF, immunofluorescence; GFAP, Glial Fibrillary Acidic Protein. Results are representative of at least three independent experiments, performed in triplicates (data points represent mean + SEM)

Increasing evidence indicates that glial cells have a role in tumor progression [9], so we investigated whether glial cells alter their secretion pattern in response to GBM cells. For that, we designed an experimental approach where primary mouse glial cell cultures were exposed to control medium or medium conditioned by a GBM murine cell line (GL261), and collected the medium from these glial cells (unprimed or primed, respectively) for subsequent proteomic and functional analyses (Fig. 1a).

We started by characterizing the glial cell cultures exposed or unexposed to GL261 CM, by evaluating their cell composition and viability. No significant differences were observed in glia metabolic cell viability (Fig. 1b), nor in cell type composition, being both conditions composed of more than 95% of GFAP-positive cells (Fig. 1c). To rule out the possibility that glial cells exposed to GBM CM could be displaying a senescence-associated secretory phenotype (SASP), that turns senescent cells into pro-inflammatory cells that have the ability to promote tumor progression (for a review [26]), we assessed the expression levels of some SASP markers [27–30]. No significant differences were detected in p21, p16, Lamin B1 and GLB1 mRNA (Additional file 3: Figure S1A) or protein levels (Additional file 3: Figure S1B) between glial cells unexposed or exposed to GBM CM, suggesting that glial cells are not undergoing senescence in response to GBM CM.

### Proteomic analyses of unprimed and primed glial cells’ CM identify key secreted proteins with potential functional impact in glioma

**Fig. 2:**
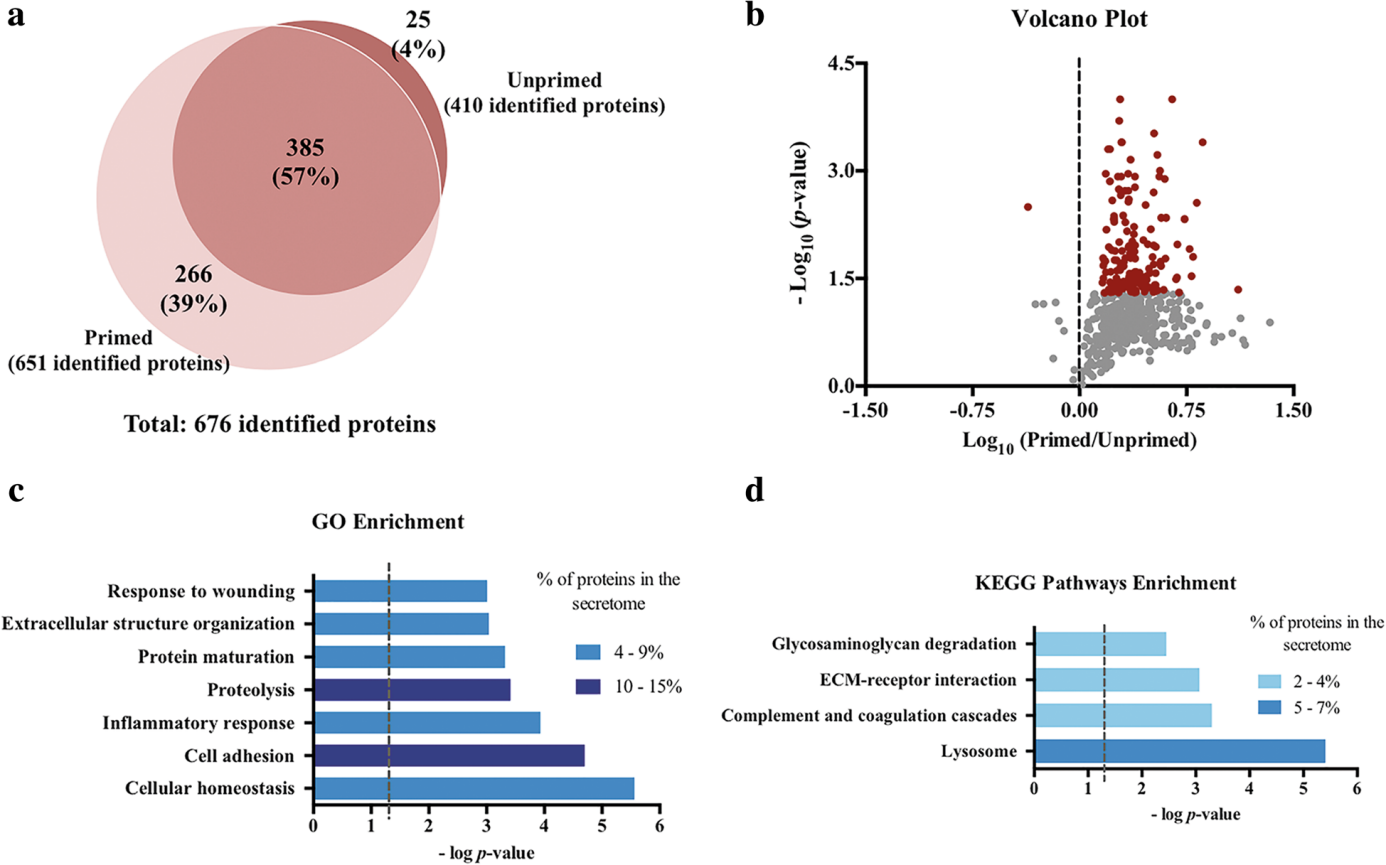
Proteomic analysis of the secretome of unprimed and primed glial cells. **a.** Venn diagram showing the total number of proteins identified in primed and unprimed glial cells CM. A higher number of proteins was identified in primed CM (651 proteins) when compared to unprimed (410 proteins), of which 385 were common to both conditions. **b.** Volcano plot of differential protein secretion in primed and unprimed glial CMs. Significantly differentially secreted proteins are colored in red. **c-d.** Database for Annotation, Visualization and Integrated Discovery (DAVID) was used to query the secretome of glial cells in order to identify enriched biological terms in the differentially secreted proteins extracted from the MS analysis. Statistically significant enriched GO terms (**c**) and KEGG pathways (**d**) are shown. Dashed lines represent *p*-value = 0.05

In order to characterize the secretome of unprimed and GBM-primed glial cells, a non-targeted systematic proteomic-based quantitative analysis was performed. Our data showed that GBM CM is able to modulate glial cells’ secretome (primed) to establish a different pattern of protein secretion when compared to the CM collected from unprimed glial cells (Fig. 2). Additionally, the pre-exposure of glial cells to GBM CM (primed condition) led to a prominent increased secretion profile of glial cells as compared to unprimed glial cells (651 proteins identified in primed glial cells vs 410 in unprimed cells, of which 385 were common to the both conditions; Fig. 2a and Additional file 4: Table S3). SWATH-MS analysis allowed the relative quantification of 534 secreted proteins. From these, 169 proteins were found up-regulated in primed glial cells and only 1 down-regulated (Fig. 2b).

To gain further insights into the biological functions of the up-regulated proteins in primed glial cells, the DAVID online annotation term enrichment tool was used (Fig. 2c-d). GO analysis showed glial cells exposed to GBM cells CM have an enrichment in proteins related to cellular homeostasis, cell adhesion, inflammatory responses, and extracellular structure organization, among others (Fig. 2c). Further analyses using KEGG integration revealed these cells’ secretome has significant enrichments for lysosome and biological processes related to complement and coagulation cascades, extracellular matrix (ECM)-receptor interaction and glycosaminoglycan degradation (Fig. 2d).

**Fig. 3:**
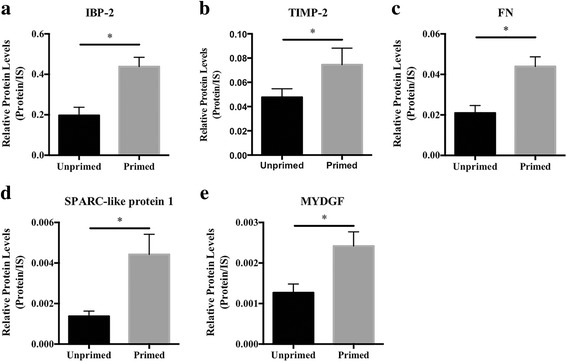
Quantitative comparison of specific secreted proteins from unprimed or primed glial cells, with putative paracrine effects in glioblastoma cells. **a.** Insulin-like growth factor-binding protein 2 (IBP-2); **b.** Metalloproteinase inhibitor 2 (TIMP-2); **c.** Fibronectin (FN); **d.** SPARC-like protein 1 and **e.** Myeloid-derived growth factor (MYDGF), were found to be upregulated in primed glial cells CM compared to unprimed CM. Values are shown as mean + SEM, *n* = 4. Statistical differences were calculated by paired Student’s t-test (*p < 0.05). IS, internal standard

Additionally, further literature mining was carried out to identify which of the secreted proteins, with potential paracrine effect, have been described to be associated with, or potentially involved in, GBM pathophysiology. Interestingly, the analysis of the relative protein levels of the two glial cells’ secretomes (unprimed and primed) for these specific proteins, allowed to find that proteins previously associated with tumoral effects, such as insulin-like growth factor-binging protein 2 (IBP-2, Fig. 3a), metalloproteinase inhibitor 2 (TIMP-2, Fig. 3b), fibronectin (FN, Fig. 3c), SPARC-like protein 1 (Fig. 3d) and myeloid-derived growth factor (MYDGF, Fig. 3e), among others, were significantly up-regulated in primed glial cells compared to unprimed. Globally, these results suggest that the priming of glia with GBM CM shifts the secretome of glia to favor a variety of tumorigenic processes.

### Functional and molecular effects of glial cells’ secretome in GBM cells

**Fig. 4:**
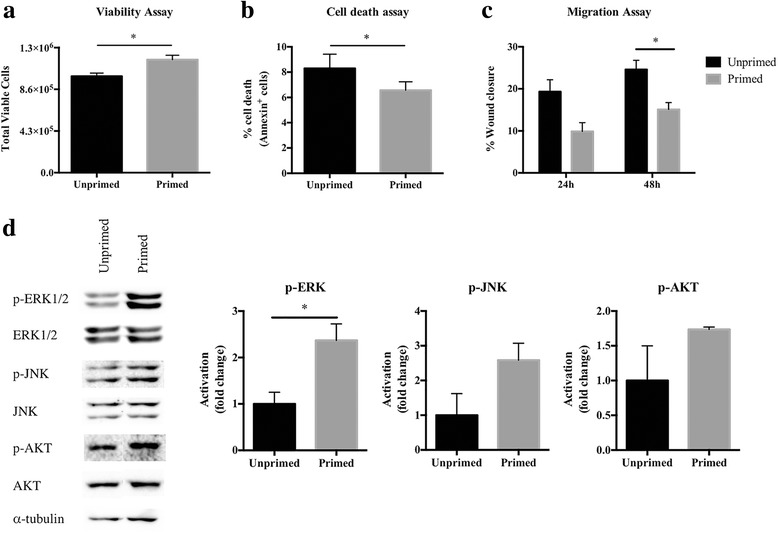
Effects of CM from unprimed or primed glial cells on GL261 GBM cells viability, cell death, and migration capacity. **a.** Cell viability trypan blue assay showing that primed CM is more efficient in promoting GBM cells viability than unprimed CM. **b.** Cell death evaluated by annexin V staining followed by flow cytometry demonstrating that primed CM decreases cell death of GL261 comparing to unprimed CM. **c.** Migration assay to evaluate cell migration capacity, which is increased in cells exposed to unprimed CM. **d.** GL261 cell lysates were analyzed by Western Blot immunostained for anti-phospho ERK, anti-phospho JNK, anti-phospho AKT, and for total ERK, JNK, AKT and α-tubulin (left). Graphs show the relative quantification of phosphorylated/total forms (right). Primed CM is able to upregulate the phosphorylation/activation of ERK pathway in GL261 cells. Results are representative of at least two independent experiments (data points represent mean + SEM). Statistical differences were calculated by paired Student’s t-test (*p < 0.05)

Our proteomics data suggests that glial cells secrete paracrine factors that might have an impact in the malignant phenotype of GBM cells. Since the factors described in Fig. 3 have been implicated in biological processes such as cell migration and invasion, cell viability and cell death, we performed a set of functional assays to investigate the phenotype of GL261 GBM cells exposed to unprimed and primed glial cells secretome. Viability assays showed that GBM cells exposed to CM from primed glial cells present increased cell viability than their unprimed counterparts (Fig. 4a). Accordingly, GBM cells exposed to primed glial cells CM present lower levels of cell death when compared to cells exposed to unprimed glial cells CM (Fig. 4b).

A major cause of GBM patients’ recurrence and resistance to therapy is the fact that GBM cells have a prominent ability to migrate throughout adjacent brain tissue. Thus, we evaluated the migration capacity of GL261 cells and the results show that they presented a significantly decreased migration capacity when exposed to primed glia, as compared to GBM cells exposed to unprimed glia CM (Fig. 4c). Together, our data showed that the priming of glial cells with GBM CM functionally modulates their secretome towards a phenotype of increased viability and cell-death resistance, while decreasing the migration capacity.

To elucidate the molecular and signaling mechanisms involved in the context-specific phenotypes observed in GBM cells exposed to unprimed or primed glial cells CM, we examined the activation of three major signaling pathways typically activated in GBM, which are known to regulate cell viability, cell death and migration capacity of cancer cells (reviewed in [31–33]): ERK, JNK and AKT. Interestingly, GL261 cells exposed to primed glial cells CM led to increased levels of ERK, JNK and AKT activation, as assessed by their phosphorylation levels (Fig. 4d). This activation was consistent in all the GL261 cells tested regardless of the batch of CM used. Nevertheless, the ratio unprimed/primed for JNK and AKT was different between batches leading to a statistically significant increase only for ERK signaling pathway (Fig. 4d, bar graphs). These molecular data at the level of the GL261 cells fits well with the differential functional effects observed in cell viability, death and migration.

## Discussion

Growing evidence of astrocytes as active participants in neuropathological conditions, such as gliomas [9] and metastatic brain tumors [34], has stimulated recent investigation into specific glial cells’ secreted proteins that may mediate these functions (reviewed in [35]). In fact, cells rarely work autonomously but rather act in concert with or in response to the cellular physiology of their neighboring cells, eliciting dynamic responses as a result of secreted signals. In the last 10 years, the secretome of murine [15, 16, 36] and human [37, 38] astrocytes has been mapped in different conditions. However, to the best of our knowledge, understanding the bidirectional communication between glial and glioma cells by characterizing the alterations occurring in the secretome of glial cells in the presence of glioma cells was still missing. Additionally, understanding the phenotypic alterations that the secretome of glial cells may have in glioma cells and the subsequent initiation of intracellular signaling events was also incomplete. Using primary cultures of mouse glial cells and GL261 glioma cells, a widely-used murine glioma model that has been shown to recapitulate the characteristics of GBM [39], we found that GBM CM led to the upregulation of several proteins in glial cells secretome (Fig. 2). This general upregulation of protein secretion is consistent with a state of astrocyte reactive gliosis, a highly heterogeneous state in which astrocytes respond to a specific injury [40]. In fact, although there are many subtypes of reactive astrogliosis, Zamanian and collaborators reported that reactive gliosis consists of a rapid induction of gene expression and identified over 1000 genes whose expression levels were induced at least two-fold in reactive astrocytes [12]. Of note, glial cells secretome after exposure to GBM cells CM was enriched in components of the extracellular matrix, cell adhesion and proteins involved in inflammatory response, partially mimicking the secretion pattern of astrocytes exposed to a cytokine cocktail for 7 days [36] or injured astrocytes [41]. Importantly, and validating our approach, approximately 80% of all the secreted proteins identified in our control condition (unprimed, Additional file 4: Table S3) are coincident with those previously identified in the CM of the control group (astrocyte cultures) of another study [16], despite the different mouse strains used (CD-1 versus C57Bl/6 in our study). Additionally, 70% of the proteins identified in another study using mass spectrometry-based proteomics and computational analysis to identify astrocytes-secreted proteins [36] were also present in our unprimed glia secretome. Concordantly, a still remarkable percentage (50%) of the secreted proteins identified in a study that analyzed astrocytes CM from a very different time-point of primary culture (8 days, versus ~20 days in our study) can also be found in our data [15]. Of note, to the best of our knowledge, all the studies reported so far mapped the secretome of pure astrocyte cultures, while we used mixed glial cultures (where the percentage of astrocytes is ~97%), which can partially explain the similarities, but also some of the differences found between our control condition and the ones previously reported.

Besides the categories above mentioned, the proteomic analysis performed in the present work also revealed many components of the astrocyte secretome that may not only regulate the composition of the extracellular matrix, but also serve as paracrine signaling hubs. Interestingly, when we tested the influence of unprimed and primed glial cells CM in GL261 glioma cells, we found that CM from primed cells promotes GBM cells viability and prevents apoptosis, contrasting with CM derived from unprimed glial cells that favors cell migration (Fig. 4). These results suggest that glial cells modulated by GBM CM, secrete factors that regulate the “go-or-grow” phenotypic switch of GBM cells, a phenomenon previously described for brain tumors, where proliferation and migration are mutually exclusive behaviors [42, 43]. Interestingly, this singularity seems to be regulated by external stimuli such as ECM components and soluble motility factors [44], suggesting that GBM cells enter a less invasive state when treated with CM from primed glial cells, mimicking GBM disease, where tumors exponentially grow in the beginning and then invade in a latter phase (i.e. cells in the tumor bulk, surrounded by activated glial cells, tend to “grow”, while cells that escape from the tumor core have a higher invasive phenotype, the “go”, by being in contact with non-activated glial cells). These effects were accompanied by the activation of ERK, AKT and JNK intracellular signaling pathways (Fig. 4d), which is in accordance with the functions of the proteins identified in this study and described to have a role as paracrine agents (Fig. 3). Indeed, IBP-2 was previously described as an inducer of proliferation in glioma cells via integrin β1/ERK signaling [45], and MYDGF is involved in cells survival as a paracrine-acting protein and also in inhibition of cell apoptosis in a PI3K/AKT-dependent signaling pathway [46, 47]. Additionally, we also found FN, a component of ECM that actively participates in cell proliferation (reviewed in [48]), to be upregulated in primed glia CM. Interestingly, FN is also described as being able to activate ERK, p38 and AKT signaling pathways [49]. On the other hand, the upregulation of SPARC-like protein 1, reported to inhibit pancreatic cancer cell invasion [50], and TIMP-2, a tissue inhibitor of metalloproteinases that inhibits endothelial cell migration [51] and reduces migration and invasion of breast cancer cells [52], fits well with the decreased migration capacity we found in GBM cells exposed to primed CM. Together, all these proteins have the potential to influence cancer cells phenotype acting in a synergistic way.

By identifying an interplay between glial cells and GBM cells, this work also opens novel opportunities for the clinical management of GBM patients. Although we do not describe the precise and complete mechanisms by which glial cells affect GBM cell viability, cell death and migration, we characterize the secretome of glial cells after exposure to GBM CM, and describe the intracellular pathways activated in GBM cells during this crosstalk. This opens a wide range of opportunities where the secretory profile of GBM cells can be therapeutically targeted to prevent this interplay with glial cells that benefits tumor progression, and thus improve the clinical management of these tumors. Further studies are warranted to identify the best targetable candidates. Additionally, the combination of these inhibitors together with specific pharmacological inhibitors of the identified intracellular signaling pathways (e.g. AKT inhibitors, revised in [53]) could be of interest in a precision medicine rationale.

## Conclusions

**Fig. 5:**
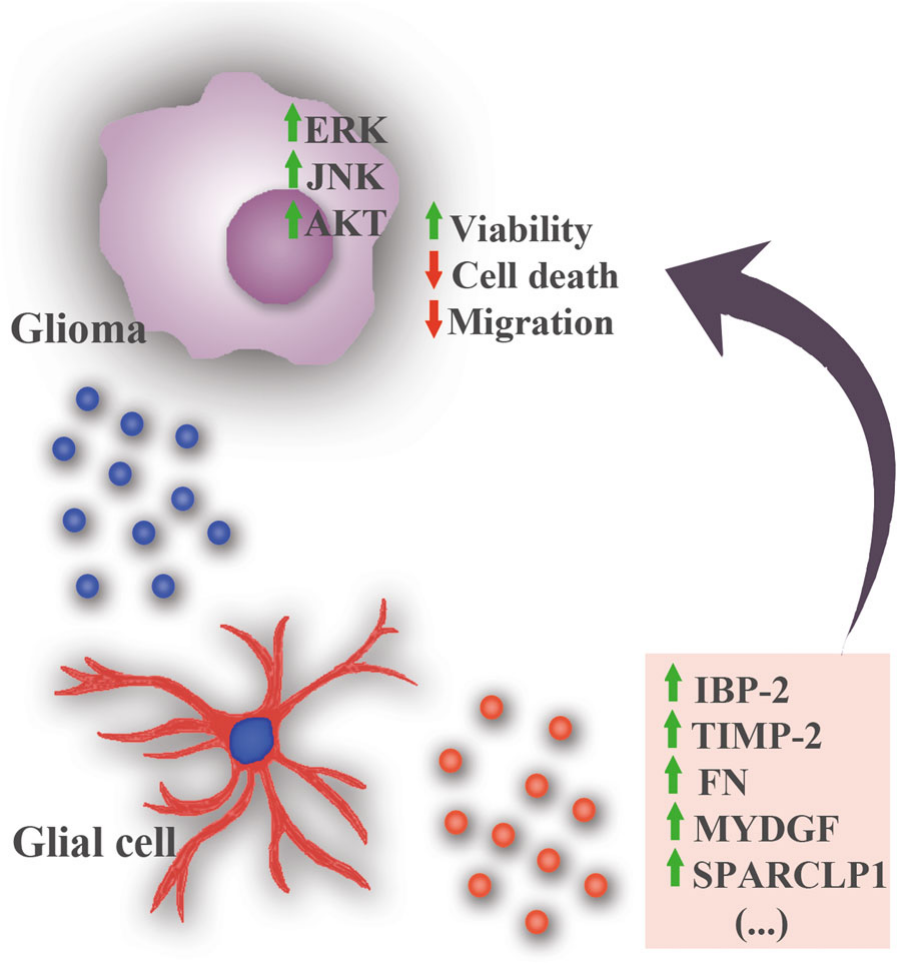
Proposed model for the molecular crosstalk between glial and glioma cells. Glioma cells secrete factors (*blue dots*) that act in a paracrine fashion to modulate glial cells. These, in turn, alter their secretome profile (*red dots*) in a way that regulates the malignant phenotype of glioma cells. For example, IBP-2, TIMP-2, FN, MYDGF and SPARCLP1 are among the upregulated proteins in the secretome of primed glial cells, which may mediate the increased viability, and the decreased cell death and migration of glioma cells through the activation of ERK, AKT and JNK signaling pathways

Our findings add new insights to the body of knowledge highlighting the relevance of the TME in gliomas and its implication for tumor progression. We found that proteins secreted by GBM cells are able to modulate glial cells that respond by secreting several proteins related to cell adhesion, extracellular matrix and inflammatory response. Moreover, we show how this modulation acts in a paracrine fashion to regulate GBM cells viability, apoptosis, and migration capacity, by regulating ERK, AKT and JNK signaling pathways (Fig. 5). In the future, validation of our major findings with additional GBM cell lines of both murine and human origin would be important to clarify if this phenomenon is universal, and how the remarkable heterogeneity typical of GBM tumors can also be explained by different interactions with the TME. Nevertheless, to study this pathway with human-derived glioma cell lines, human glial cells are required to avoid false negative/positive interactions due to species-specific differences in protein composition/structure. By characterizing the dynamic process of glial cells secretion in the presence/absence of tumor cells, and showing that these findings play an important role in GBM progression, this study also contributes to the rational development of novel combinatory antitumor strategies to treat malignant gliomas.

## Additional files

Additional file 1: Table S1. Sequence of primers used for quantitative RT-PCR analyses. (DOCX 19 kb)

Additional file 2: Table S2. SWATH-MS variable windows used in the acquisition of the samples used for pull-down analysis. For each window is indicated the *m/z* range, the window width in Dalton (Da) and the CES. (DOCX 19 kb)

Additional file 3: Figure S1. Glial cells exposed to GBM CM do not change the expression of senescence-associated secretory phenotype markers. **a.** mRNA expression levels of *p16*, *GLB1*, *Lamin B1* and *p21* assessed by qPCR showing that there are no significant differences in the transcriptional levels of these genes between glial cells unexposed and exposed to GBM CM. **b.** Western Blot immunostaining for anti-p16, anti-GLB1, anti-Lamin B1 and anti-p21 in glial cells (left). Graph shows the relative quantification based on the total intensity of the sample loaded (right). No significant differences are found between unexposed and exposed glial cells. Abbreviations: U, unexposed; E, exposed. Results are representative of two independent experiments (data points represent mean + SEM). Statistical differences were calculated by paired Student’s t-test. (TIFF 2081 kb)

Additional file 4: Table S3. Proteins identified in pooled samples of unprimed and primed CM. (DOCX 56 kb)

## Abbreviations

ACM: Astrocytes-derived conditioned media
CES: Collision energy spread
CM: Conditioned media
CNS: Central nervous system
DAVID: Database for Annotation, Visualization and Integrated Discovery
DMEM: Dulbecco’s Modified Eagle Medium
ECM: Extracellular matrix
FBS: Fetal bovine serum
FDR: False discovery rate
FN: Fibronectin
GBM: Glioblastoma
GFAP: Glial Fibrillary Acidic Protein
GO: Gene Ontology
IBP-2: Insulin-like growth factor-binding protein 2
ID: Internal diameter
IDA: Information-dependent acquisition
IF: Immunofluorescence
KEGG: Kyoto Encyclopedia of Genes and Genomes
MS: Mass spectrometry
MYDGF: Myeloid-derived growth factor
ON: Overnight
PRIDE: Proteomics Identifications
RT: Room temperature
SASP: Senescence-Associated Secretory Phenotype
TCA: Trichloroacetic acid
TIMP-2: Metalloproteinase inhibitor 2
TME: Tumor microenvironment
TMZ: Temozolomide
XIC: Extracted-ion chromatogram
