# Supplementary material for: Crosstalk between glial and glioblastoma cells triggers the “go-or-grow” phenotype of tumor cells
Source: Cell Commun Signal. 2017 Oct 2;15:37. doi: 10.1186/s12964-017-0194-x (PMC5625790; doi:10.1186/s12964-017-0194-x)
Supplement: Supplementary file 1 — Sequence of primers used for quantitative RT-PCR analyses. (DOCX 19 kb) [file 12964_2017_194_MOESM1_ESM.docx]

Table S1. Sequence of primers used for quantitative RT-PCR analyses.

| **Gene** | **Primer Sense** | **Primer Antisense** | **Primer**  **Tm (°C)** | **Product size (bp)** |
| --- | --- | --- | --- | --- |
| ***p21*** | CGAGAACGGTGGAACTTTGAC | CAGGGCTCAGGTAGACCTTG | 64 | 106 |
| ***p16*** | GAACTCTTTCGGTCGTACCC | TGGGCGTGCTTGAGCTGA | 60 | 368 |
| ***Lamin B1*** | CAGGAATTGGAGGACATGCT | GAAGGGCTTGGAGAGAGCTT | 62 | 221 |
| ***GLB1*** | CACTGCTGCAACTGCTGG | ATGTATCGGAATGGCTGTCC | 59 | 115 |
| ***TBP*** | GGGAGAATCATGGACCAGAA | TTGCTGCTGCTGTCTTTGTT | 55 | 192 |

For p21 gene, qPCR parameters were as follow: 2 minutes at 50 °C, 2 minutes at 95 °C, 40 cycles of denaturation for 15 seconds at 95 °C, annealing for 30 seconds, and final extension increasing the temperature in 1°C each 5 seconds from 65°C to 95°C. For all the other genes, qPCR parameters were as follows: 4 minutes at 94 °C, 40 cycles of denaturation for 30 seconds at 94 °C, annealing for 30 seconds, and extension at 72 °C for 30 seconds, and final extension increasing the temperature in 1°C each 5 seconds from 65°C to 95°C.
