# Supplementary material for: Crosstalk between glial and glioblastoma cells triggers the “go-or-grow” phenotype of tumor cells
Source: Cell Commun Signal. 2017 Oct 2;15:37. doi: 10.1186/s12964-017-0194-x (PMC5625790; doi:10.1186/s12964-017-0194-x)
Supplement: Supplementary file 2 — SWATH-MS variable windows used in the acquisition of the samples used for pull-down analysis. For each window is indicated the m/z range, the window width in Dalton (Da) and the CES. (DOCX 19 kb) [file 12964_2017_194_MOESM2_ESM.docx]

Table S2. SWATH-MS variable windows used in the acquisition of the samples used for pull-down analysis. For each window is indicated the *m/z* range, the window width in Dalton (Da) and the CES.

|  | *m/z* range | Width (Da) | CES |
| --- | --- | --- | --- |
| Window 1 | 349.5-360 | 10.5 | 5 |
| Window 2 | 359-369 | 10 | 5 |
| Window 3 | 368-377.9 | 9.9 | 5 |
| Window 4 | 376.9-386.5 | 9.6 | 5 |
| Window 5 | 385.5-395 | 9.5 | 5 |
| Window 6 | 394-403.6 | 9.6 | 5 |
| Window 7 | 402.6-412.1 | 9.5 | 5 |
| Window 8 | 411.1-420.7 | 9.6 | 5 |
| Window 9 | 419.7-429.2 | 9.5 | 5 |
| Window 10 | 428.2-437.3 | 9.1 | 5 |
| Window 11 | 436.3-445.9 | 9.6 | 5 |
| Window 12 | 444.9-454 | 9.1 | 5 |
| Window 13 | 453-461.6 | 8.6 | 5 |
| Window 14 | 460.6-469.7 | 9.1 | 5 |
| Window 15 | 468.7-477.4 | 8.7 | 5 |
| Window 16 | 476.4-485.5 | 9.1 | 5 |
| Window 17 | 484.5-493.1 | 8.6 | 5 |
| Window 18 | 492.1-501.2 | 9.1 | 5 |
| Window 19 | 500.2-508.9 | 8.7 | 5 |
| Window 20 | 507.9-516.5 | 8.6 | 5 |
| Window 21 | 515.5-524.2 | 8.7 | 5 |
| Window 22 | 523.2-532.3 | 9.1 | 5 |
| Window 23 | 531.3-539.9 | 8.6 | 5 |
| Window 24 | 538.9-547.6 | 8.7 | 5 |
| Window 25 | 546.6-554.8 | 8.2 | 5 |
| Window 26 | 553.8-562.5 | 8.7 | 5 |
| Window 27 | 561.5-570.1 | 8.6 | 5 |
| Window 28 | 569.1-577.3 | 8.2 | 5 |
| Window 29 | 576.3-585.4 | 9.1 | 5 |
| Window 30 | 584.4-593.5 | 9.1 | 5 |
| Window 31 | 592.5-601.2 | 8.7 | 5 |
| Window 32 | 600.2-608.8 | 8.6 | 5 |
| Window 33 | 607.8-616.9 | 9.1 | 5 |
| Window 34 | 615.9-625.5 | 9.6 | 5 |
| Window 35 | 624.5-633.6 | 9.1 | 5 |
| Window 36 | 632.6-642.6 | 10 | 5 |
| Window 37 | 641.6-651.1 | 9.5 | 5 |
| Window 38 | 650.1-660.1 | 10 | 5 |
| Window 39 | 659.1-670 | 10.9 | 5 |
| Window 40 | 669-680.4 | 11.4 | 5 |
| Window 41 | 679.4-691.2 | 11.8 | 5 |
| Window 42 | 690.2-703.3 | 13.1 | 5 |
| Window 43 | 702.3-715 | 12.7 | 5 |
| Window 44 | 714-727.6 | 13.6 | 5 |
| Window 45 | 726.6-739.8 | 13.2 | 5 |
| Window 46 | 738.8-752.4 | 13.6 | 5 |
| Window 47 | 751.4-764.5 | 13.1 | 5 |
| Window 48 | 763.5-778 | 14.5 | 5 |
| Window 49 | 777-791.5 | 14.5 | 5 |
| Window 50 | 790.5-805.9 | 15.4 | 8 |
| Window 51 | 804.9-822.1 | 17.2 | 8 |
| Window 52 | 821.1-839.2 | 18.1 | 8 |
| Window 53 | 838.2-859.5 | 21.3 | 8 |
| Window 54 | 858.5-882 | 23.5 | 8 |
| Window 55 | 881-906.7 | 25.7 | 8 |
| Window 56 | 905.7-937.3 | 31.6 | 8 |
| Window 57 | 936.3-976.5 | 40.2 | 10 |
| Window 58 | 975.5-1021.9 | 46.4 | 10 |
| Window 59 | 1020.9-1098.9 | 78 | 10 |
| Window 60 | 1097.9-1249.6 | 151.7 | 5 |
