# Supplementary figures and images for: Crosstalk between glial and glioblastoma cells triggers the “go-or-grow” phenotype of tumor cells
Source: Cell Commun Signal. 2017 Oct 2;15:37. doi: 10.1186/s12964-017-0194-x (PMC5625790; doi:10.1186/s12964-017-0194-x)

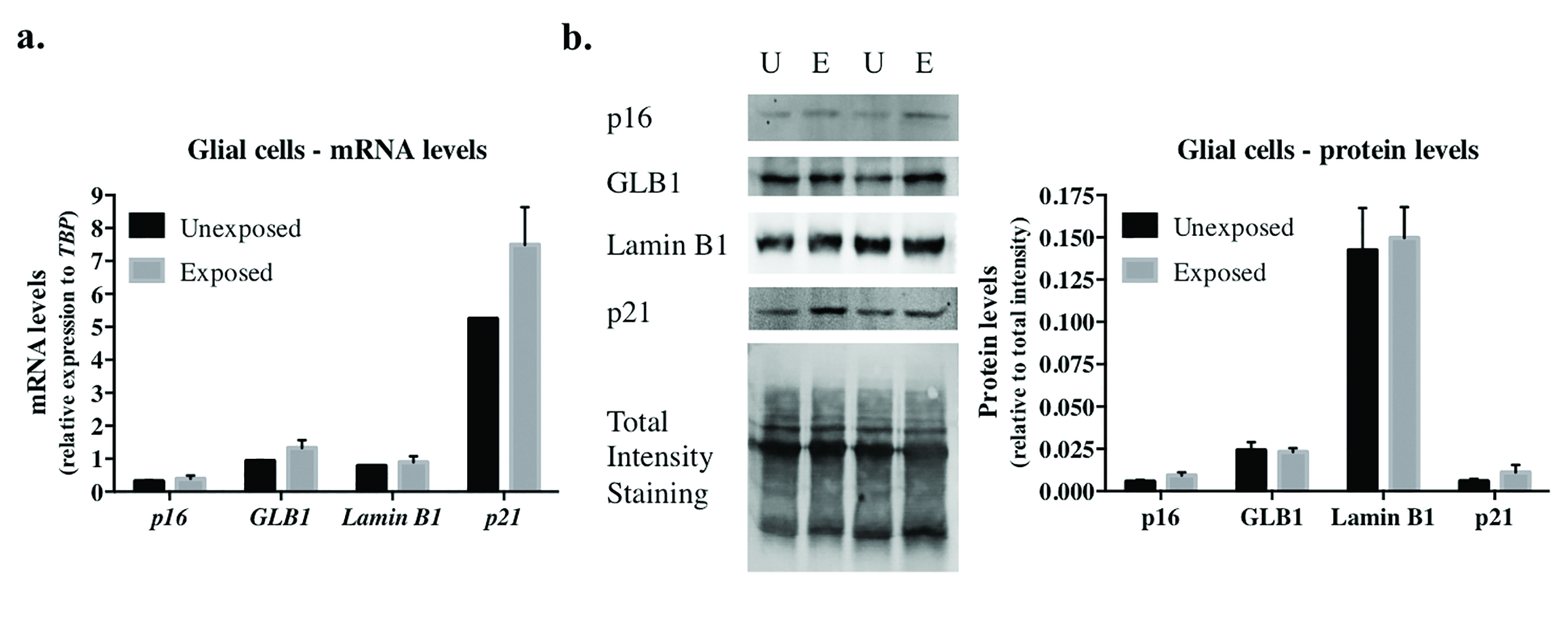

Supplement: Supplementary file 3 — Glial cells exposed to GBM CM do not change the expression of senescence-associated secretory phenotype markers. a. mRNA expression levels of p16, GLB1, Lamin B1 and p21 assessed by qPCR showing that there are no significant differences in the transcriptional levels of these genes between glial cells unexposed and exposed to GBM CM. b. Western Blot immunostaining for anti-p16, anti-GLB1, anti-Lamin B1 and anti-p21 in glial cells (left). Graph shows the relative quantification based on the total intensity of the sample loaded (right). No significant differences are found between unexposed and exposed glial cells. Abbreviations: U, unexposed; E, exposed. Results are representative of two independent experiments (data points represent mean + SEM). Statistical differences were calculated by paired Student’s t-test. (TIFF 2081 kb) [file 12964_2017_194_MOESM3_ESM.tif]
