# Supplementary material for: Crosstalk between glial and glioblastoma cells triggers the “go-or-grow” phenotype of tumor cells
Source: Cell Commun Signal. 2017 Oct 2;15:37. doi: 10.1186/s12964-017-0194-x (PMC5625790; doi:10.1186/s12964-017-0194-x)
Supplement: Supplementary file 4 — Proteins identified in pooled samples of unprimed and primed CM. (DOCX 56 kb) [file 12964_2017_194_MOESM4_ESM.docx]

**Table S3.** Proteins identified in pooled samples of unprimed and primed CM.

| **UniProt ID** | **Protein name** | **Condition (unprimed/primed)** |
| --- | --- | --- |
| Q64433 | 10 kDa heat shock protein | Both |
| Q9CQV8 | 14-3-3 protein beta/alpha | Both |
| P62259 | 14-3-3 protein epsilon | Both |
| P68510 | 14-3-3 protein eta | Both |
| P61982 | 14-3-3 protein gamma | Both |
| P68254 | 14-3-3 protein theta | Both |
| P63101 | 14-3-3 protein zeta/delta | Both |
| O88685 | 26S protease regulatory subunit 6A | Primed |
| Q3TXS7 | 26S proteasome non-ATPase regulatory subunit 1 | Primed |
| O35593 | 26S proteasome non-ATPase regulatory subunit 14 | Primed |
| P14685 | 26S proteasome non-ATPase regulatory subunit 3 | Primed |
| Q8BJY1 | 26S proteasome non-ATPase regulatory subunit 5 | Both |
| Q9CX56 | 26S proteasome non-ATPase regulatory subunit 8 | Primed |
| Q8BWT1 | 3-ketoacyl-CoA thiolase | Unprimed |
| P63325 | 40S ribosomal protein S10 | Both |
| P14131 | 40S ribosomal protein S16 | Unprimed |
| P60867 | 40S ribosomal protein S20 | Primed |
| P62267 | 40S ribosomal protein S23 | Primed |
| P62858 | 40S ribosomal protein S28 | Both |
| P62908 | 40S ribosomal protein S3 | Both |
| P62702 | 40S ribosomal protein S4 | Primed |
| P62082 | 40S ribosomal protein S7 | Unprimed |
| P14206 | 40S ribosomal protein SA | Primed |
| Q61112 | 45 kDa calcium-binding protein | Both |
| P10852 | 4F2 cell-surface antigen heavy chain | Both |
| Q9DCD0 | 6-phosphogluconate dehydrogenase | Both |
| Q9CQ60 | 6-phosphogluconolactonase | Both |
| P63038 | 60 kDa heat shock protein | Primed |
| P14869 | 60S acidic ribosomal protein P0 | Primed |
| P47955 | 60S acidic ribosomal protein P1 | Primed |
| P62889 | 60S ribosomal protein L30 | Primed |
| Q9JJI8 | 60S ribosomal protein L38 | Primed |
| P47962 | 60S ribosomal protein L5 | Primed |
| P33434 | 72 kDa type IV collagenase | Primed |
| P20029 | 78 kDa glucose-regulated protein | Primed |
| P97857 | A disintegrin and metalloproteinase with thrombospondin motifs 1 | Primed |
| Q9WTQ5 | A-kinase anchor protein 12 | Both |
| Q9WV54 | Acid ceramidase | Both |
| P70158 | Acid sphingomyelinase-like phosphodiesterase 3a | Both |
| Q99KI0 | Aconitate hydratase | Primed |
| P61161 | Actin-related protein 2 | Primed |
| Q9CVB6 | Actin-related protein 2/3 complex subunit 2 | Primed |
| Q9JM76 | Actin-related protein 2/3 complex subunit 3 | Both |
| P59999 | Actin-related protein 2/3 complex subunit 4 | Both |
| Q9CPW4 | Actin-related protein 2/3 complex subunit 5 | Both |
| Q99JY9 | Actin-related protein 3 | Primed |
| P62737 | Actin, aortic smooth muscle | Both |
| P63260 | Actin, cytoplasmic 2 | Both |
| P31786 | Acyl-CoA-binding protein | Both |
| Q9QYR9 | Acyl-coenzyme A thioesterase 2 | Both |
| P56376 | Acylphosphatase-1 | Both |
| Q64010 | Adapter molecule crk | Primed |
| Q9WTP6 | Adenylate kinase 2 | Both |
| Q9R0Y5 | Adenylate kinase isoenzyme 1 | Primed |
| P40124 | Adenylyl cyclase-associated protein 1 | Primed |
| P84078 | ADP-ribosylation factor 1 | Primed |
| P84084 | ADP-ribosylation factor 5 | Unprimed |
| Q9WUL7 | ADP-ribosylation factor-like protein 3 | Primed |
| O89020 | Afamin | Primed |
| Q9JII6 | Alcohol dehydrogenase [NADP | Both |
| P28474 | Alcohol dehydrogenase class-3 | Both |
| P47738 | Aldehyde dehydrogenase, mitochondrial | Both |
| Q8VC28 | Aldo-keto reductase family 1 member C13 | Primed |
| P45376 | Aldose reductase | Both |
| Q8R4G6 | Alpha-1,6-mannosylglycoprotein 6-beta-N-acetylglucosaminyltransferase A | Both |
| P29699 | Alpha-2-HS-glycoprotein | Primed |
| Q6GQT1 | Alpha-2-macroglobulin-P | Both |
| Q7TPR4 | Alpha-actinin-1 | Both |
| P57780 | Alpha-actinin-4 | Both |
| Q9DBF1 | Alpha-aminoadipic semialdehyde dehydrogenase | Primed |
| P17182 | Alpha-enolase | Both |
| Q8VCT3 | Aminopeptidase B | Both |
| P12023 | Amyloid beta A4 protein | Both |
| P09470 | Angiotensin-converting enzyme | Primed |
| P10107 | Annexin A1 | Primed |
| P07356 | Annexin A2 | Both |
| O35639 | Annexin A3 | Both |
| P48036 | Annexin A5 | Both |
| P14824 | Annexin A6 | Primed |
| P32261 | Antithrombin-III | Both |
| P17426 | AP-2 complex subunit alpha-1 | Primed |
| Q9DBG3 | AP-2 complex subunit beta | Primed |
| Q00623 | Apolipoprotein A-I | Primed |
| P08226 | Apolipoprotein E | Both |
| Q07813 | Apoptosis regulator BAX | Primed |
| P16460 | Argininosuccinate synthase | Both |
| P50428 | Arylsulfatase A | Both |
| P50429 | Arylsulfatase B | Both |
| P05201 | Aspartate aminotransferase, cytoplasmic | Both |
| P05202 | Aspartate aminotransferase, mitochondrial | Both |
| Q922B2 | Aspartate--tRNA ligase, cytoplasmic | Primed |
| Q9Z2W0 | Aspartyl aminopeptidase | Primed |
| Q62048 | Astrocytic phosphoprotein PEA-15 | Both |
| P28658 | Ataxin-10 | Unprimed |
| Q03265 | ATP synthase subunit alpha, mitochondrial | Both |
| P56480 | ATP synthase subunit beta, mitochondrial | Both |
| Q99LS0 | Augurin | Both |
| Q05793 | Basement membrane-specific heparan sulfate proteoglycan core protein | Primed |
| P15209 | BDNF/NT-3 growth factors receptor | Both |
| O09010 | Beta-1,3-N-acetylglucosaminyltransferase lunatic fringe | Both |
| Q8BWP8 | Beta-1,4-glucuronyltransferase 1 | Both |
| P01887 | Beta-2-microglobulin | Both |
| Q8R5C5 | Beta-centractin | Primed |
| P23780 | Beta-galactosidase | Both |
| P12265 | Beta-glucuronidase | Both |
| P29416 | Beta-hexosaminidase subunit alpha | Both |
| P20060 | Beta-hexosaminidase subunit beta | Both |
| Q9CWJ9 | Bifunctional purine biosynthesis protein PURH | Both |
| P28653 | Biglycan | Both |
| Q8CIF4 | Biotinidase | Both |
| Q8BTJ4 | Bis(5'-adenosyl)-triphosphatase enpp4 | Primed |
| Q8R016 | Bleomycin hydrolase | Both |
| P98063 | Bone morphogenetic protein 1 | Primed |
| P20722 | Bone morphogenetic protein 6 | Primed |
| Q61361 | Brevican core protein | Primed |
| P17515 | C-X-C motif chemokine 10 | Both |
| P15116 | Cadherin-2 | Both |
| P39038 | Cadherin-4 | Primed |
| Q06138 | Calcium-binding protein 39 | Primed |
| P62204 | Calmodulin | Primed |
| Q08091 | Calponin-1 | Primed |
| Q08093 | Calponin-2 | Primed |
| Q9DAW9 | Calponin-3 | Both |
| P14211 | Calreticulin | Primed |
| Q9EPL2 | Calsyntenin-1 | Both |
| O35887 | Calumenin | Unprimed |
| Q80WV3 | Carbohydrate sulfotransferase 2 | Primed |
| P00920 | Carbonic anhydrase 2 | Primed |
| P18761 | Carbonic anhydrase 6 | Primed |
| Q00493 | Carboxypeptidase E | Both |
| Q9WVJ3 | Carboxypeptidase Q | Both |
| Q02248 | Catenin beta-1 | Both |
| P10605 | Cathepsin B | Both |
| P18242 | Cathepsin D | Both |
| P06797 | Cathepsin L1 | Both |
| Q8BM88 | Cathepsin O | Both |
| O70370 | Cathepsin S | Both |
| Q9WUU7 | Cathepsin Z | Both |
| Q8R422 | CD109 antigen | Both |
| P15379 | CD44 antigen | Both |
| P35762 | CD81 antigen | Primed |
| Q8R5M8 | Cell adhesion molecule 1 | Both |
| Q99N28 | Cell adhesion molecule 3 | Unprimed |
| Q8R464 | Cell adhesion molecule 4 | Both |
| P60766 | Cell division control protein 42 homolog | Both |
| Q8R2Y2 | Cell surface glycoprotein MUC18 | Primed |
| P53996 | Cellular nucleic acid-binding protein | Primed |
| Q61147 | Ceruloplasmin | Both |
| Q61362 | Chitinase-3-like protein 1 | Both |
| Q9Z1Q5 | Chloride intracellular channel protein 1 | Both |
| Q9QYB1 | Chloride intracellular channel protein 4 | Primed |
| Q9CZU6 | Citrate synthase, mitochondrial | Both |
| Q61548 | Clathrin coat assembly protein AP180 | Primed |
| Q68FD5 | Clathrin heavy chain 1 | Both |
| Q6IRU5 | Clathrin light chain B | Primed |
| Q06890 | Clusterin | Both |
| Q9CQI6 | Coactosin-like protein | Both |
| Q9QZE5 | Coatomer subunit gamma-1 | Primed |
| P18760 | Cofilin-1 | Both |
| P45591 | Cofilin-2 | Both |
| P11087 | Collagen alpha-1 | Primed |
| P39061 | Collagen alpha-1 | Primed |
| Q00780 | Collagen alpha-1 | Both |
| Q04857 | Collagen alpha-1 | Primed |
| Q61245 | Collagen alpha-1 | Both |
| P08121 | Collagen alpha-1 (III) chain | Primed |
| P02463 | Collagen alpha-1 (IV) chain | Both |
| Q60847 | Collagen alpha-1(XII) chain | Both |
| Q01149 | Collagen alpha-2 | Both |
| Q3U962 | Collagen alpha-2 | Both |
| P08122 | Collagen alpha-2 (IV) chain | Both |
| Q64739 | Collagen alpha-2 (XI) chain | Primed |
| P98086 | Complement C1q subcomponent subunit A | Both |
| P14106 | Complement C1q subcomponent subunit B | Both |
| Q8K479 | Complement C1q tumor necrosis factor-related protein 5 | Both |
| Q8CG16 | Complement C1r-A subcomponent | Both |
| Q8CG14 | Complement C1s-A subcomponent | Both |
| P01027 | Complement C3 | Both |
| P01029 | Complement C4-B | Both |
| P29268 | Connective tissue growth factor | Both |
| O88544 | COP9 signalosome complex subunit 4 | Primed |
| O08997 | Copper transport protein ATOX1 | Primed |
| Q9WUM4 | Coronin-1C | Both |
| Q4KML4 | Costars family protein ABRACL | Primed |
| Q04447 | Creatine kinase B-type | Both |
| Q62426 | Cystatin-B | Both |
| P21460 | Cystatin-C | Both |
| P97315 | Cysteine and glycine-rich protein 1 | Both |
| P56391 | Cytochrome c oxidase subunit 6B1 | Both |
| P62897 | Cytochrome c, somatic | Primed |
| P28271 | Cytoplasmic aconitate hydratase | Primed |
| Q9CPY7 | Cytosol aminopeptidase | Both |
| Q8R0Y6 | Cytosolic 10-formyltetrahydrofolate dehydrogenase | Both |
| Q91V12 | Cytosolic acyl coenzyme A thioester hydrolase | Primed |
| Q9D1A2 | Cytosolic non-specific dipeptidase | Primed |
| Q61753 | D-3-phosphoglycerate dehydrogenase | Primed |
| O35215 | D-dopachrome decarboxylase | Primed |
| Q8JZM4 | Delta and Notch-like epidermal growth factor-related receptor | Unprimed |
| Q9R0P5 | Destrin | Both |
| Q8R242 | Di-N-acetylchitobiase | Both |
| O08749 | Dihydrolipoyl dehydrogenase, mitochondrial | Both |
| Q8BVI4 | Dihydropteridine reductase | Primed |
| O08553 | Dihydropyrimidinase-related protein 2 | Both |
| Q62188 | Dihydropyrimidinase-related protein 3 | Primed |
| Q9ET22 | Dipeptidyl peptidase 2 | Both |
| Q99KK7 | Dipeptidyl peptidase 3 | Primed |
| O35598 | Disintegrin and metalloproteinase domain-containing protein 10 | Primed |
| Q61072 | Disintegrin and metalloproteinase domain-containing protein 9 | Primed |
| Q3U1J4 | DNA damage-binding protein 1 | Both |
| P28352 | DNA-(apurinic or apyrimidinic site) lyase | Primed |
| Q99KJ8 | Dynactin subunit 2 | Primed |
| Q62165 | Dystroglycan | Both |
| Q9EQG7 | Ectonucleotide pyrophosphatase/phosphodiesterase family member 5 | Both |
| Q9D8Y0 | EF-hand domain-containing protein D2 | Both |
| Q8BPB5 | EGF-containing fibulin-like extracellular matrix protein 1 | Both |
| Q9WVJ9 | EGF-containing fibulin-like extracellular matrix protein 2 | Both |
| P70372 | ELAV-like protein 1 | Primed |
| P10126 | Elongation factor 1-alpha 1 | Both |
| Q9D8N0 | Elongation factor 1-gamma | Primed |
| P58252 | Elongation factor 2 | Both |
| P57759 | Endoplasmic reticulum resident protein 29 | Both |
| Q9D1Q6 | Endoplasmic reticulum resident protein 44 | Primed |
| P08113 | Endoplasmin | Primed |
| Q8BGB7 | Enolase-phosphatase E1 | Primed |
| P42125 | Enoyl-CoA delta isomerase 1, mitochondrial | Both |
| Q03137 | Ephrin type-A receptor 4 | Both |
| P54763 | Ephrin type-B receptor 2 | Primed |
| Q9Z0J0 | Epididymal secretory protein E1 | Both |
| O54782 | Epididymis-specific alpha-mannosidase | Both |
| Q9D379 | Epoxide hydrolase 1 | Primed |
| O70258 | Epsilon-sarcoglycan | Primed |
| Q8CHU3 | Epsin-2 | Primed |
| Q91V76 | Ester hydrolase C11orf54 homolog | Both |
| P60843 | Eukaryotic initiation factor 4A-I | Primed |
| P63242 | Eukaryotic translation initiation factor 5A-1 | Primed |
| P56564 | Excitatory amino acid transporter 1 | Primed |
| P97464 | Exostosin-1 | Primed |
| P70428 | Exostosin-2 | Primed |
| Q9ES89 | Exostosin-like 2 | Primed |
| Q61508 | Extracellular matrix protein 1 | Both |
| O09164 | Extracellular superoxide dismutase | Both |
| P26040 | Ezrin | Both |
| P47754 | F-actin-capping protein subunit alpha-2 | Both |
| P47757 | F-actin-capping protein subunit beta | Both |
| Q80UW2 | F-box only protein 2 | Both |
| Q920E5 | Farnesyl pyrophosphate synthase | Primed |
| Q61553 | Fascin | Both |
| P51880 | Fatty acid-binding protein, brain | Both |
| P50608 | Fibromodulin | Both |
| P11276 | Fibronectin | Both |
| Q9WVH9 | Fibulin-5 | Both |
| Q8BTM8 | Filamin-A | Both |
| Q80X90 | Filamin-B | Both |
| Q8VHX6 | Filamin-C | Primed |
| Q62356 | Follistatin-related protein 1 | Both |
| P97447 | Four and a half LIM domains protein 1 | Both |
| O35188 | Fractalkine | Both |
| P05064 | Fructose-bisphosphate aldolase A | Both |
| P05063 | Fructose-bisphosphate aldolase C | Both |
| Q8R2K1 | Fucose mutarotase | Primed |
| P97807 | Fumarate hydratase, mitochondrial | Primed |
| P54818 | Galactocerebrosidase | Primed |
| P16045 | Galectin-1 | Both |
| P16110 | Galectin-3 | Both |
| Q07797 | Galectin-3-binding protein | Both |
| Q9Z0L8 | Gamma-glutamyl hydrolase | Both |
| Q60648 | Ganglioside GM2 activator | Both |
| P23591 | GDP-L-fucose synthase | Primed |
| P13020 | Gelsolin | Both |
| Q9CQI3 | Glia maturation factor beta | Primed |
| Q07235 | Glia-derived nexin | Both |
| P03995 | Glial fibrillary acidic protein | Both |
| P06745 | Glucose-6-phosphate isomerase | Both |
| O08795 | Glucosidase 2 subunit beta | Primed |
| P26443 | Glutamate dehydrogenase 1, mitochondrial | Both |
| P15105 | Glutamine synthetase | Primed |
| Q9CQM9 | Glutaredoxin-3 | Both |
| P47791 | Glutathione reductase, mitochondrial | Both |
| P24472 | Glutathione S-transferase A4 | Both |
| P10649 | Glutathione S-transferase Mu 1 | Both |
| P15626 | Glutathione S-transferase Mu 2 | Primed |
| P48774 | Glutathione S-transferase Mu 5 | Primed |
| P19157 | Glutathione S-transferase P 1 | Both |
| P51855 | Glutathione synthetase | Primed |
| P16858 | Glyceraldehyde-3-phosphate dehydrogenase | Both |
| Q8CI94 | Glycogen phosphorylase, brain form | Both |
| Q9CPV4 | Glyoxalase domain-containing protein 4 | Primed |
| Q91Z53 | Glyoxylate reductase/hydroxypyruvate reductase | Both |
| P51655 | Glypican-4 | Both |
| P28798 | Granulins | Both |
| Q01721 | Growth arrest-specific protein 1 | Primed |
| Q61592 | Growth arrest-specific protein 6 | Primed |
| P62827 | GTP-binding nuclear protein Ran | Unprimed |
| Q9WTP7 | GTP:AMP phosphotransferase AK3, mitochondrial | Primed |
| P18872 | Guanine nucleotide-binding protein G | Primed |
| P62880 | Guanine nucleotide-binding protein G | Primed |
| Q3V3I2 | Guanine nucleotide-binding protein G | Unprimed |
| P01899 | H-2 class I histocompatibility antigen, D-B alpha chain | Primed |
| P14426 | H-2 class I histocompatibility antigen, D-K alpha chain | Both |
| Q3UGR5 | Haloacid dehalogenase-like hydrolase domain-containing protein 2 | Both |
| Q61316 | Heat shock 70 kDa protein 4 | Both |
| P63017 | Heat shock cognate 71 kDa protein | Both |
| P07901 | Heat shock protein HSP 90-alpha | Primed |
| P11499 | Heat shock protein HSP 90-beta | Both |
| P01942 | Hemoglobin subunit alpha | Primed |
| P02089 | Hemoglobin subunit beta-2 | Both |
| Q99020 | Heterogeneous nuclear ribonucleoprotein A/B | Primed |
| P49312 | Heterogeneous nuclear ribonucleoprotein A1 | Primed |
| Q60668 | Heterogeneous nuclear ribonucleoprotein D0 | Primed |
| Q9Z2X1 | Heterogeneous nuclear ribonucleoprotein F | Primed |
| P61979 | Heterogeneous nuclear ribonucleoprotein K | Both |
| Q7TMK9 | Heterogeneous nuclear ribonucleoprotein Q | Primed |
| O88569 | Heterogeneous nuclear ribonucleoproteins A2/B1 | Both |
| Q9Z204 | Heterogeneous nuclear ribonucleoproteins C1/C2 | Primed |
| P17710 | Hexokinase-1 | Primed |
| P70349 | Histidine triad nucleotide-binding protein 1 | Primed |
| Q61035 | Histidine--tRNA ligase, cytoplasmic | Primed |
| P43277 | Histone H1.3 | Primed |
| Q64522 | Histone H2A type 2-B | Unprimed |
| Q8R1M2 | Histone H2A.J | Primed |
| Q8CGP2 | Histone H2B type 1-P | Primed |
| P84244 | Histone H3.3 | Primed |
| P62806 | Histone H4 | Both |
| Q60973 | Histone-binding protein RBBP7 | Primed |
| Q61425 | Hydroxyacyl-coenzyme A dehydrogenase, mitochondrial | Both |
| Q6GU68 | Immunoglobulin superfamily containing leucine-rich repeat protein | Primed |
| Q9EQS9 | Immunoglobulin superfamily DCC subclass member 4 | Primed |
| P70168 | Importin subunit beta-1 | Primed |
| Q8BKG3 | Inactive tyrosine-protein kinase 7 | Both |
| Q9D819 | Inorganic pyrophosphatase | Primed |
| O55023 | Inositol monophosphatase 1 | Primed |
| P47877 | Insulin-like growth factor-binding protein 2 | Both |
| P47878 | Insulin-like growth factor-binding protein 3 | Both |
| P47879 | Insulin-like growth factor-binding protein 4 | Primed |
| Q07079 | Insulin-like growth factor-binding protein 5 | Both |
| Q61581 | Insulin-like growth factor-binding protein 7 | Both |
| Q80W15 | Insulin-like growth factor-binding protein-like 1 | Unprimed |
| O89051 | Integral membrane protein 2B | Both |
| Q91VK4 | Integral membrane protein 2C | Primed |
| Q62470 | Integrin alpha-3 | Primed |
| A6X935 | Inter alpha-trypsin inhibitor, heavy chain 4 | Unprimed |
| Q61703 | Inter-alpha-trypsin inhibitor heavy chain H2 | Both |
| Q61704 | Inter-alpha-trypsin inhibitor heavy chain H3 | Primed |
| Q8BJD1 | Inter-alpha-trypsin inhibitor heavy chain H5 | Unprimed |
| O35664 | Interferon alpha/beta receptor 2 | Both |
| Q64345 | Interferon-induced protein with tetratricopeptide repeats 3 | Primed |
| Q61730 | Interleukin-1 receptor accessory protein | Primed |
| Q8C0M9 | Isoaspartyl peptidase/L-asparaginase | Both |
| Q9D6R2 | Isocitrate dehydrogenase [NAD] subunit alpha, mitochondrial | Primed |
| O88844 | Isocitrate dehydrogenase [NADP] cytoplasmic | Both |
| P58044 | Isopentenyl-diphosphate Delta-isomerase 1 | Both |
| Q61897 | Keratin, type I cuticular Ha3-II | Unprimed |
| P02535 | Keratin, type I cytoskeletal 10 | Both |
| Q9QWL7 | Keratin, type I cytoskeletal 17 | Both |
| Q9ERE2 | Keratin, type II cuticular Hb1 | Unprimed |
| Q99M74 | Keratin, type II cuticular Hb2 | Unprimed |
| P04104 | Keratin, type II cytoskeletal 1 | Both |
| Q6IFZ6 | Keratin, type II cytoskeletal 1b | Both |
| Q3TTY5 | Keratin, type II cytoskeletal 2 epidermal | Both |
| Q3UV17 | Keratin, type II cytoskeletal 2 oral | Primed |
| Q922U2 | Keratin, type II cytoskeletal 5 | Both |
| P50446 | Keratin, type II cytoskeletal 6A | Unprimed |
| Q9Z331 | Keratin, type II cytoskeletal 6B | Primed |
| Q8VED5 | Keratin, type II cytoskeletal 79 | Both |
| P11679 | Keratin, type II cytoskeletal 8 | Unprimed |
| P06151 | L-lactate dehydrogenase A chain | Both |
| P16125 | L-lactate dehydrogenase B chain | Both |
| P21956 | Lactadherin | Primed |
| Q9CPU0 | Lactoylglutathione lyase | Both |
| P14733 | Lamin-B1 | Primed |
| O08999 | Latent-transforming growth factor beta-binding protein 2 | Both |
| P70202 | Latexin | Both |
| O89017 | Legumain | Both |
| P0C192 | Leucine-rich repeat-containing protein 4B | Primed |
| P24527 | Leukotriene A-4 hydrolase | Both |
| Q61792 | LIM and SH3 domain protein 1 | Both |
| Q99PU5 | Long-chain-fatty-acid--CoA ligase ACSBG1 | Primed |
| Q9D358 | Low molecular weight phosphotyrosine protein phosphatase | Both |
| P35951 | Low-density lipoprotein receptor | Primed |
| Q99MN1 | Lysine--tRNA ligase | Primed |
| P24638 | Lysosomal acid phosphatase | Primed |
| O09159 | Lysosomal alpha-mannosidase | Both |
| P16675 | Lysosomal protective protein | Both |
| P11438 | Lysosome-associated membrane glycoprotein 1 | Both |
| P17047 | Lysosome-associated membrane glycoprotein 2 | Both |
| P08905 | Lysozyme C-2 | Both |
| Q9Z175 | Lysyl oxidase homolog 3 | Both |
| P07141 | Macrophage colony-stimulating factor 1 | Both |
| P09581 | Macrophage colony-stimulating factor 1 receptor | Primed |
| P34884 | Macrophage migration inhibitory factor | Primed |
| P24452 | Macrophage-capping protein | Both |
| P04925 | Major prion protein | Primed |
| P14152 | Malate dehydrogenase, cytoplasmic | Both |
| P08249 | Malate dehydrogenase, mitochondrial | Both |
| Q99M71 | Mammalian ependymin-related protein 1 | Both |
| P45700 | Mannosyl-oligosaccharide 1,2-alpha-mannosidase IA | Both |
| Q9DBV4 | Matrix-remodeling-associated protein 8 | Both |
| Q61865 | Melanoma-derived growth regulatory protein | Both |
| Q9CXI5 | Mesencephalic astrocyte-derived neurotrophic factor | Both |
| P12032 | Metalloproteinase inhibitor 1 | Primed |
| P25785 | Metalloproteinase inhibitor 2 | Both |
| P39876 | Metalloproteinase inhibitor 3 | Primed |
| Q8C1Q4 | Meteorin | Both |
| Q8VE43 | Meteorin-like protein | Both |
| P27546 | Microtubule-associated protein 4 | Both |
| Q61166 | Microtubule-associated protein RP/EB family member 1 | Primed |
| Q62000 | Mimecan | Both |
| P10404 | MLV-related proviral Env polyprotein | Primed |
| P26041 | Moesin | Both |
| P10810 | Monocyte differentiation antigen CD14 | Both |
| Q6DIB5 | Multiple epidermal growth factor-like domains protein 10 | Both |
| Q9Z2L6 | Multiple inositol polyphosphate phosphatase 1 | Both |
| Q9CPT4 | Myeloid-derived growth factor | Both |
| Q60605 | Myosin light polypeptide 6 | Both |
| Q3THE2 | Myosin regulatory light chain 12B | Primed |
| Q8VDD5 | Myosin-9 | Both |
| P62774 | Myotrophin | Primed |
| Q571E4 | N-acetylgalactosamine-6-sulfatase | Both |
| Q8BFR4 | N-acetylglucosamine-6-sulfatase | Both |
| Q64191 | N(4)-(beta-N-acetylglucosaminyl)-L-asparaginase | Both |
| Q9CWS0 | N(G),N(G)-dimethylarginine dimethylaminohydrolase 1 | Both |
| Q8K4Z3 | NAD(P)H-hydrate epimerase | Both |
| P06801 | NADP-dependent malic enzyme | Both |
| P70670 | Nascent polypeptide-associated complex subunit alpha, muscle-specific form | Primed |
| P97798 | Neogenin | Primed |
| Q6P5H2 | Nestin | Primed |
| P13595 | Neural cell adhesion molecule 1 | Both |
| Q61477 | Neuroblastoma suppressor of tumorigenicity 1 | Primed |
| P55066 | Neurocan core protein | Both |
| Q810U4 | Neuronal cell adhesion molecule | Primed |
| Q80Z24 | Neuronal growth regulator 1 | Both |
| P35803 | Neuronal membrane glycoprotein M6-b | Primed |
| P97300 | Neuroplastin | Primed |
| O35684 | Neuroserpin | Both |
| Q8BHN3 | Neutral alpha-glucosidase AB | Both |
| Q8CCH2 | NHL repeat-containing protein 3 | Both |
| O88322 | Nidogen-2 | Both |
| O88998 | Noelin | Primed |
| Q8BM13 | Noelin-2 | Primed |
| Q02819 | Nucleobindin-1 | Both |
| P09405 | Nucleolin | Primed |
| P15532 | Nucleoside diphosphate kinase A | Primed |
| Q01768 | Nucleoside diphosphate kinase B | Both |
| Q9JHW2 | Omega-amidase NIT2 | Both |
| P29758 | Ornithine aminotransferase, mitochondrial | Both |
| O35103 | Osteomodulin | Both |
| Q8QZR4 | Out at first protein homolog | Both |
| O88531 | Palmitoyl-protein thioesterase 1 | Both |
| Q8CI51 | PDZ and LIM domain protein 5 | Both |
| P97467 | Peptidyl-glycine alpha-amidating monooxygenase | Both |
| P17742 | Peptidyl-prolyl cis-trans isomerase A | Both |
| P24369 | Peptidyl-prolyl cis-trans isomerase B | Both |
| P30412 | Peptidyl-prolyl cis-trans isomerase C | Both |
| P45878 | Peptidyl-prolyl cis-trans isomerase FKBP2 | Primed |
| Q9Z247 | Peptidyl-prolyl cis-trans isomerase FKBP9 | Primed |
| P35700 | Peroxiredoxin-1 | Both |
| Q61171 | Peroxiredoxin-2 | Both |
| O08807 | Peroxiredoxin-4 | Primed |
| P99029 | Peroxiredoxin-5, mitochondrial | Both |
| O08709 | Peroxiredoxin-6 | Both |
| P70296 | Phosphatidylethanolamine-binding protein 1 | Both |
| P53810 | Phosphatidylinositol transfer protein alpha isoform | Both |
| Q9D0F9 | Phosphoglucomutase-1 | Primed |
| P09411 | Phosphoglycerate kinase 1 | Both |
| Q9DBJ1 | Phosphoglycerate mutase 1 | Both |
| P55065 | Phospholipid transfer protein | Both |
| Q99K85 | Phosphoserine aminotransferase | Both |
| P97298 | Pigment epithelium-derived factor | Both |
| P97290 | Plasma protease C1 inhibitor | Both |
| P22777 | Plasminogen activator inhibitor 1 | Both |
| Q61233 | Plastin-2 | Primed |
| Q99K51 | Plastin-3 | Both |
| Q60963 | Platelet-activating factor acetylhydrolase | Both |
| Q61206 | Platelet-activating factor acetylhydrolase IB subunit beta | Both |
| Q925I7 | Platelet-derived growth factor D | Both |
| Q6PE55 | Platelet-derived growth factor receptor-like protein | Primed |
| P20033 | Platelet-derived growth factor subunit A | Primed |
| Q9QXS1 | Plectin | Primed |
| Q9DC11 | Plexin domain-containing protein 2 | Both |
| Q8CJH3 | Plexin-B1 | Primed |
| B2RXS4 | Plexin-B2 | Both |
| P60335 | Poly(rC)-binding protein 1 | Both |
| Q61990 | Poly(rC)-binding protein 2 | Both |
| P29341 | Polyadenylate-binding protein 1 | Primed |
| Q9JJ61 | Polypeptide N-acetylgalactosaminyltransferase 16 | Both |
| Q6PB93 | Polypeptide N-acetylgalactosaminyltransferase 2 | Primed |
| P17225 | Polypyrimidine tract-binding protein 1 | Primed |
| P48678 | Prelamin-A/C | Both |
| Q6NSR8 | Probable aminopeptidase NPEPL1 | Primed |
| Q8BHA3 | Probable D-tyrosyl-tRNA | Primed |
| Q61398 | Procollagen C-endopeptidase enhancer 1 | Both |
| Q9R0E2 | Procollagen-lysine,2-oxoglutarate 5-dioxygenase 1 | Both |
| Q9R0B9 | Procollagen-lysine,2-oxoglutarate 5-dioxygenase 2 | Both |
| Q9R0E1 | Procollagen-lysine,2-oxoglutarate 5-dioxygenase 3 | Primed |
| P62962 | Profilin-1 | Both |
| Q9JJV2 | Profilin-2 | Primed |
| P17918 | Proliferating cell nuclear antigen | Primed |
| Q91ZX7 | Prolow-density lipoprotein receptor-related protein 1 | Both |
| Q9QUR6 | Prolyl endopeptidase | Primed |
| Q9QXV0 | ProSAAS | Both |
| Q61207 | Prosaposin | Both |
| Q8VDQ1 | Prostaglandin reductase 2 | Unprimed |
| O09114 | Prostaglandin-H2 D-isomerase | Both |
| P97371 | Proteasome activator complex subunit 1 | Primed |
| P49722 | Proteasome subunit alpha type-2 | Primed |
| O70435 | Proteasome subunit alpha type-3 | Primed |
| Q9R1P0 | Proteasome subunit alpha type-4 | Both |
| Q9Z2U1 | Proteasome subunit alpha type-5 | Both |
| Q9QUM9 | Proteasome subunit alpha type-6 | Both |
| Q9Z2U0 | Proteasome subunit alpha type-7 | Both |
| O09061 | Proteasome subunit beta type-1 | Both |
| Q9R1P3 | Proteasome subunit beta type-2 | Both |
| Q9R1P1 | Proteasome subunit beta type-3 | Both |
| P99026 | Proteasome subunit beta type-4 | Primed |
| O55234 | Proteasome subunit beta type-5 | Primed |
| Q60692 | Proteasome subunit beta type-6 | Primed |
| Q9JIF0 | Protein arginine N-methyltransferase 1 | Both |
| O88668 | Protein CREG1 | Primed |
| Q80YA8 | Protein crumbs homolog 2 | Both |
| Q99LX0 | Protein deglycase DJ-1 | Both |
| P09103 | Protein disulfide-isomerase | Primed |
| P27773 | Protein disulfide-isomerase A3 | Both |
| Q922R8 | Protein disulfide-isomerase A6 | Both |
| Q91VU0 | Protein FAM3C | Primed |
| E9Q7X6 | Protein HEG homolog 1 | Both |
| P08207 | Protein S100-A10 | Unprimed |
| P50543 | Protein S100-A11 | Both |
| Q9CX34 | Protein SGT1 homolog | Primed |
| P23492 | Purine nucleoside phosphorylase | Both |
| Q11011 | Puromycin-sensitive aminopeptidase | Both |
| Q3TCN2 | Putative phospholipase B-like 2 | Both |
| Q8K183 | Pyridoxal kinase | Both |
| P52480 | Pyruvate kinase PKM | Both |
| P50396 | Rab GDP dissociation inhibitor alpha | Both |
| Q61598 | Rab GDP dissociation inhibitor beta | Both |
| Q9JKF1 | Ras GTPase-activating-like protein IQGAP1 | Primed |
| P62492 | Ras-related protein Rab-11A | Primed |
| P62821 | Ras-related protein Rab-1A | Primed |
| P59279 | Ras-related protein Rab-2B | Primed |
| Q99JI6 | Ras-related protein Rap-1b | Primed |
| P68040 | Receptor of activated protein C kinase 1 | Both |
| Q05909 | Receptor-type tyrosine-protein phosphatase gamma | Both |
| P28828 | Receptor-type tyrosine-protein phosphatase mu | Primed |
| B9EKR1 | Receptor-type tyrosine-protein phosphatase zeta | Both |
| Q9CYN9 | Renin receptor | Primed |
| Q8BP92 | Reticulocalbin-2 | Both |
| Q99P72 | Reticulon-4 | Primed |
| P24549 | Retinal dehydrogenase 1 | Both |
| Q9DD06 | Retinoic acid receptor responder protein 2 | Both |
| Q920A5 | Retinoid-inducible serine carboxypeptidase | Both |
| Q99PT1 | Rho GDP-dissociation inhibitor 1 | Both |
| Q9JJH1 | Ribonuclease 4 | Both |
| Q91VI7 | Ribonuclease inhibitor | Primed |
| Q9CQ01 | Ribonuclease T2 | Primed |
| P52760 | Ribonuclease UK114 | Both |
| Q9D7G0 | Ribose-phosphate pyrophosphokinase 1 | Primed |
| Q8R2Q4 | Ribosome-releasing factor 2, mitochondrial | Both |
| Q9JI75 | Ribosyldihydronicotinamide dehydrogenase | Both |
| Q91VM5 | RNA binding motif protein, X-linked-like-1 | Primed |
| Q9WV02 | RNA-binding motif protein, X chromosome | Unprimed |
| O89086 | RNA-binding protein 3 | Primed |
| Q9WTM5 | RuvB-like 2 | Primed |
| Q3THS6 | S-adenosylmethionine synthase isoform type-2 | Primed |
| Q9R0P3 | S-formylglutathione hydrolase | Both |
| Q9WTX5 | S-phase kinase-associated protein 1 | Primed |
| O88745 | Scrapie-responsive protein 1 | Primed |
| Q9CZC8 | Secernin-1 | Both |
| P47867 | Secretogranin-3 | Both |
| Q7TSK2 | Seizure protein 6 | Primed |
| Q62181 | Semaphorin-3C | Unprimed |
| Q64105 | Sepiapterin reductase | Primed |
| P42208 | Septin-2 | Primed |
| Q8CHH9 | Septin-8 | Primed |
| Q80UG5 | Septin-9 | Primed |
| Q9D6X6 | Serine protease 23 | Both |
| Q9R118 | Serine protease HTRA1 | Both |
| Q91WP6 | Serine protease inhibitor A3N | Both |
| Q76MZ3 | Serine/threonine-protein phosphatase 2A 65 kDa regulatory subunit A alpha isoform | Primed |
| P58389 | Serine/threonine-protein phosphatase 2A activator | Primed |
| P62141 | Serine/threonine-protein phosphatase PP1-beta catalytic subunit | Primed |
| Q921I1 | Serotransferrin | Both |
| Q60854 | Serpin B6 | Both |
| P19324 | Serpin H1 | Both |
| P07724 | Serum albumin | Both |
| Q9JJU8 | SH3 domain-binding glutamic acid-rich-like protein | Both |
| P62305 | Small nuclear ribonucleoprotein E | Primed |
| P62320 | Small nuclear ribonucleoprotein Sm D3 | Primed |
| Q9Z172 | Small ubiquitin-related modifier 3 | Primed |
| Q8VCF1 | Soluble calcium-activated nucleotidase 1 | Both |
| Q62417 | Sorbin and SH3 domain-containing protein 1 | Primed |
| Q3UTJ2 | Sorbin and SH3 domain-containing protein 2 | Both |
| Q64442 | Sorbitol dehydrogenase | Both |
| Q6PHU5 | Sortilin | Primed |
| O88307 | Sortilin-related receptor | Primed |
| O70492 | Sorting nexin-3 | Primed |
| P07214 | SPARC | Both |
| P70663 | SPARC-like protein 1 | Primed |
| P16546 | Spectrin alpha chain, non-erythrocytic 1 | Both |
| Q62261 | Spectrin beta chain, non-erythrocytic 1 | Primed |
| Q64674 | Spermidine synthase | Primed |
| Q9Z1N5 | Spliceosome RNA helicase Ddx39b | Primed |
| P26369 | Splicing factor U2AF 65 kDa subunit | Primed |
| Q8VIJ6 | Splicing factor, proline- and glutamine-rich | Primed |
| Q8VCC9 | Spondin-1 | Both |
| Q60598 | Src substrate cortactin | Both |
| P38647 | Stress-70 protein, mitochondrial | Primed |
| Q8K2B3 | Succinate dehydrogenase [ubiquinone] flavoprotein subunit, mitochondrial | Both |
| Q9D0K2 | Succinyl-CoA:3-ketoacid coenzyme A transferase 1, mitochondrial | Both |
| Q8BND5 | Sulfhydryl oxidase 1 | Both |
| P08228 | Superoxide dismutase [Cu-Zn] | Both |
| P09671 | Superoxide dismutase [Mn] | Both |
| O08992 | Syntenin-1 | Primed |
| P11983 | T-complex protein 1 subunit alpha | Primed |
| P80314 | T-complex protein 1 subunit beta | Both |
| P80315 | T-complex protein 1 subunit delta | Primed |
| P80316 | T-complex protein 1 subunit epsilon | Primed |
| P80313 | T-complex protein 1 subunit eta | Both |
| P80318 | T-complex protein 1 subunit gamma | Both |
| P42932 | T-complex protein 1 subunit theta | Primed |
| P26039 | Talin-1 | Both |
| Q80YX1 | Tenascin | Both |
| Q8C1A5 | Thimet oligopeptidase | Primed |
| P10639 | Thioredoxin | Both |
| Q9CQU0 | Thioredoxin domain-containing protein 12 | Primed |
| Q91W90 | Thioredoxin domain-containing protein 5 | Both |
| Q9JMH6 | Thioredoxin reductase 1 | Both |
| P20108 | Thioredoxin-dependent peroxide reductase | Both |
| P35441 | Thrombospondin-1 | Both |
| P01831 | Thy-1 membrane glycoprotein | Both |
| Q99LJ1 | Tissue alpha-L-fucosidase | Both |
| Q93092 | Transaldolase | Both |
| O88968 | Transcobalamin-2 | Both |
| P37804 | Transgelin | Both |
| Q9WVA4 | Transgelin-2 | Primed |
| Q01853 | Transitional endoplasmic reticulum ATPase | Both |
| P40142 | Transketolase | Both |
| P63028 | Translationally-controlled tumor protein | Both |
| Q62348 | Translin | Primed |
| P07309 | Transthyretin | Both |
| P17751 | Triosephosphate isomerase | Both |
| Q9Z0L0 | Trophoblast glycoprotein | Primed |
| P58771 | Tropomyosin alpha-1 chain | Primed |
| Q6IRU2 | Tropomyosin alpha-4 chain | Primed |
| P68369 | Tubulin alpha-1A chain | Both |
| P05213 | Tubulin alpha-1B chain | Unprimed |
| P68368 | Tubulin alpha-4A chain | Primed |
| Q9CWF2 | Tubulin beta-2B chain | Both |
| P68372 | Tubulin beta-4B chain | Both |
| P99024 | Tubulin beta-5 chain | Both |
| Q91YR1 | Twinfilin-1 | Primed |
| P56399 | Ubiquitin carboxyl-terminal hydrolase 5 | Both |
| Q9R0P9 | Ubiquitin carboxyl-terminal hydrolase isozyme L1 | Both |
| P62984 | Ubiquitin-60S ribosomal protein L40 | Both |
| P61079 | Ubiquitin-conjugating enzyme E2 D3 | Primed |
| P61087 | Ubiquitin-conjugating enzyme E2 K | Primed |
| P68037 | Ubiquitin-conjugating enzyme E2 L3 | Both |
| P61089 | Ubiquitin-conjugating enzyme E2 N | Both |
| Q9D2M8 | Ubiquitin-conjugating enzyme E2 variant 2 | Primed |
| Q02053 | Ubiquitin-like modifier-activating enzyme 1 | Both |
| Q3TW96 | UDP-N-acetylhexosamine pyrophosphorylase-like protein 1 | Primed |
| Q9DBP5 | UMP-CMP kinase | Primed |
| Q91ZJ5 | UTP--glucose-1-phosphate uridylyltransferase | Both |
| P54728 | UV excision repair protein RAD23 homolog B | Unprimed |
| Q9R1Q9 | V-type proton ATPase subunit S1 | Both |
| Q9EQH3 | Vacuolar protein sorting-associated protein 35 | Primed |
| Q8R164 | Valacyclovir hydrolase | Primed |
| P29533 | Vascular cell adhesion protein 1 | Both |
| Q9DBH5 | Vesicular integral-membrane protein VIP36 | Primed |
| P20152 | Vimentin | Both |
| Q64727 | Vinculin | Both |
| Q08761 | Vitamin K-dependent protein S | Primed |
| P29788 | Vitronectin | Primed |
| Q99KC8 | von Willebrand factor A domain-containing protein 5A | Both |
| O88342 | WD repeat-containing protein 1 | Both |
| Q11136 | Xaa-Pro dipeptidase | Both |
| Q811B1 | Xylosyltransferase 1 | Primed |
